# Supplementary material for: Variation in health visiting contacts for children in England: cross-sectional analysis of the 2–2½ year review using administrative data (Community Services Dataset, CSDS)
Source: BMJ Open. 2022 Feb 22;12(2):e053884. doi: 10.1136/bmjopen-2021-053884 (PMC8867374; doi:10.1136/bmjopen-2021-053884)
Supplement: Supplementary data [file bmjopen-2021-053884supp001.pdf]

Supplementary Material 1: Identifying a 'research-ready' subset of CSDS for use in research into the 2-2½ year review

The material in supplementary material 1 and 2 is based on our report: https://www.ucl.ac.uk/children-policy-research/sites/children-policy-research/files/using_csds_for_research_report_08.10.20.pdf

Methods

Identifying "high correlation" data for local authorities

To identify local authorities within CSDS with sufficiently complete data for research into 2-2½ year reviews, we created two indicators of how well data was recorded in CSDS and sense-checked these indicators with a Consultant in Public Health working with a Health Visiting Service (SB), an information analyst who manages health visiting data (GW) and the lead for PHE metrics (KT). The indicators measure the agreement between CSDS and aggregate reference data (PHE metrics or ONS births). Based on these indicators, we categorised local authorities as having high, moderate or low correlation between data in CSDS and reference data.

The indicators are based on the number of eligible children and children who had 2-2½ year reviews recorded (Table A1). These indicators were selected to account for the two mechanisms of under-recording in CSDS that would affect research into health visiting activity. First, some children may be missing completely from the data. If children are missing from CSDS, we do not have accurate data on how many children are eligible for health visiting and therefore do not have an accurate denominator to calculate the percentage of children who received contacts. We were able to determine whether the number of eligible children were accurate through comparisons between CSDS and ONS birth statistics. We focused on "eligible children" who were aged 0 because we did not have reliable reference data on the number of children aged 2 or 3 to compare with CSDS. Second, contacts with health visitors may be under-recorded. We were able to determine whether the number of contacts were accurate through comparisons between CSDS and PHE metrics.

Table A1: The indicators and limits for identifying high or moderate correlation local authorities in CSDS

Indicator	Limits	
	**High correlation**	**Moderate correlation**
**(i) Eligible children:** Agreement between the number of children aged 0 at the end of the reporting year in CSDS and ONS birth statistics	- 10% to +20%	-30% to +30%
**(ii) 2-2.5 year reviews:** Agreement between the number of children with a 2-2.5 year review in CSDS and PHE metrics	-15% to +15%	-40% to +40%

We used local data from East Sussex and Kent to select appropriate limits for the high and moderate correlation categories, by comparing local data to the reference data. The percentage difference between the local data and PHE metrics ranged from -10% to +1% for the new birth visit and 2-2½ year review (See Table A1). Based on this, we considered a difference between CSDS and PHE

metrics of +/-15% to be appropriate for high correlation and +/-40% appropriate for moderate correlation. We expect the true number of "eligible children" (children born in 2018/19) to be higher than the number of children born in England, because CSDS should include children born elsewhere who moved to England before their first birthday. This was true in our comparisons of local data and ONS births, where there were 3-8% more children aged <1 year in the local data compared to ONS births. This is reflected in the limits we selected for high correlation for eligible children (-10% to +20%).

Local authorities that did not meet either high or moderate criteria were classified as low correlation. As for many local authorities agreement with the indicators varied by quarter, we allowed local authorities to move between categories by quarter. This means that data from specific time periods within a local authority could be considered as high correlation and therefore included in analyses. The contact date was used to allocate children who had a contact to a local authority quarter (and therefore to a correlation category). For children without contacts (i.e. to calculate the denominator population), we could not determine which child became eligible in each quarter. Therefore, we calculated the total number of children per local authority divided by four to estimate the number of children per quarter.

Are children in the high correlation local authorities nationally representative?

To draw conclusions from research using the subset of children in the high correlation group we must understand whether results can be extrapolated to children in other local authorities in CSDS, and to all children whether or not they were recorded in CSDS. To determine whether the subset of local authorities were representative, we focused on the IMD quintile and ethnicity because of the completeness of these variables in CSDS (IMD is complete for all children in the study and ethnicity is complete for 82% of children) and because there were a substantial number of children within each IMD or ethnicity category.

To determine whether the chances of a child being recorded in CSDS varied by IMD quintile and ethnicity, we compared the distribution of IMD and ethnicity in ONS births to children in CSDS who were born in 2018/19. We also compared the distribution of IMD quintile and ethnicity of children aged <5 years in CSDS and local data for two local authorities (East Sussex and Barking and Dagenham). We did not have data from 2018/19 from Barking and Dagenham, therefore we compared 2017/18 local data to 2018/19 CSDS data. We do not expect any significant changes in the distribution of IMD and ethnicity in such a short time frame (one year). To determine whether the high correlation group was representative of the whole of CSDS, we compared the ethnicity and IMD in the high correlation group to all of CSDS for children aged less than 5 years.

Results

Identifying "high correlation" local authorities

Thirty-three local authorities met the high correlation criteria for research into the 2-2½ year review. These are listed in Box 1. These groupings do not reflect the quality of local data recording for case management purposes or the quality of the health visiting service. The groupings are based solely on the correlation between the reference data and the CSDS extract.

Box 1: Local authorities that had data in CSDS with high correlation between data in CSDS and reference data based on indicators in table 2 for 2018/19

Blackpool	Hertfordshire	Sefton
Calderdale	Islington	Shropshire
Cornwall and Isles of Scilly	Kingston upon Thames	Somerset
Coventry	Kirklees	Southampton
Cumbria	Knowsley	St Helens
Derby	Lincolnshire	Telford and Wrekin
Devon	Liverpool	Thurrock
Dudley	North Somerset	Walsall
Gloucestershire	Peterborough	Warrington
Hampshire	Portsmouth	Worcestershire
Haringey	Rochdale	York

Are children in the high correlation local authorities nationally representative?

Recording of children in CSDS varied by ethnicity but not deprivation. ONS births reported a higher percentage of births of White children compared to CSDS: 72% vs 61% (Table A2). However, the data from ONS births included Wales, which likely has a higher proportion of White children than England. The percentage of children who were Asian, Black or another ethnicity were similar, but 14% of children were missing ethnicity compared to 4% in ONS births. This suggests that White children in CSDS were more likely to have missing data for ethnicity. In East Sussex, White children were under-represented and children with mixed ethnicity were over-represented in CSDS, compared to local data. In Barking and Dagenham, the percentage of White children was similar in CSDS and local data, but the local data recorded more children with mixed ethnicity and fewer Black and Asian children. As the overall number of children was similar, this is likely due to differences in recording, rather than differences in which children are in the datasets. The distribution of IMD in CSDS was similar to ONS births and local data from East Sussex and Barking and Dagenham (Table A6).

Children in the high correlation group were not representative of all children in CSDS in terms of ethnicity. The percentage of children in the mixed, Asian, Black and other ethnicity categories was lower in the high correlation group compared to all of CSDS (Table A2). This likely reflects regional differences in ethnicity. People in the Black, Asian, mixed and other ethnic groups are more likely to live in London than any other region in England, but only three of 32 London boroughs are in the high correlation subset(31). Children in the high correlation group were similar in terms of deprivation.

Table A2: The distribution of ethnicity and IMD for children aged less than 5 in the high correlation group compared to all CSDS and children in ONS births compared to births in CSDS

	Children aged <5 years in 2018/19		Births	
	Children in the high correlation group (33 LAs) [1]	All children in CSDS (149 LAs)	Children in ONS births (2018) [2]	Children in CSDS born in 2018/19 (151 LAs)
**Ethnicity**				
White	297,365 (68%)	1,845,171 (56%)	468,456 (72%)	316,820 (61%)
Mixed	32,255 (7%)	325,145 (10%)	[3]	[3]
Asian	23,350 (5%)	298,071 (9%)	56,821 (8%)	48,265 (9%)
Black	9,865 (2%)	129,352 (4%)	27,363 (4%)	19,610 (4%)
Other	10,200 (2%)	109,987 (3%)	77,060 (12%)	59,880 (12%)
Missing	64,360 (15%)	609,939 (18%)	27,023 (4%)	75,375 (14%)
Total	437,395	3,314,665	656,723	519,950
**IMD**				
1 (most deprived)	112,305 (26%)	832,885 (25%)	161,709 (26%)	129,915 (25%)
2	88,285 (20%)	714,728 (22%)	140,416 (22%)	113,005 (22%)
3	85,145 (19%)	640,986 (19%)	121,500 (19%)	103,655 (20%)
4	76,630 (18%)	575,712 (17%)	107,339 (17%)	91,340 (18%)
5 (least deprived)	75,025 (17%)	550,354 (17%)	94,687 (15%)	82,030 (16%)
Total	437,390	3,314,665	625,651	519,945

[1] CSDS data for the high correlation LAs is rounded to the nearest 5 [2] Ethnicity includes children born in Wales which is why the total is higher; [3] ONS do not report mixed as an ethnicity category in ONS births, therefore we have grouped mixed with other for this comparison

Supplementary Material 2: Estimating the percentage of eligible children with a 2-2½ year review in CSDS

We were unable to determine time at risk or the exact number of children in CSDS who were eligible for the 2-2½ year review, due to the lack of a precise measure of age in our CSDS extract. The age variable available was age on 31st March 2019. Children eligible for the 2-2½ year review during 2018/19 could be aged 2 or 3 at the end of 2018/19, and we could not identify those children who received their review during 2017/18 or 2019/20.

To estimate the percentage of children who had a 2-2½ year review, we used the number of children who had the 2-2½ year review and were aged 2 (at end of 2018/19) as the numerator (Equation 1). To calculate the denominator, we used all children aged 2 (at the end of 2018/19) minus the number of children who had the 2-2½ year review aged 3 (at end of 2018/19). This was based on the assumptions that (i) children who had the 2-2½ year review and were aged 3 (at end of 2018/19) were born towards the end of the year (i.e. were younger than 2½ years at the start of 2018/19), and (ii) a similar number of children who were aged 2 (at end of 2018/19) would have not yet turned 2½ years and would be expected to have their 2-2½ year review in 2019/20. If we had not estimated the percentage in this way, we would have either only counted children who had their 2-2½ year review and were aged 2 at the end of 2018/19 (excluding children who were born late in the year and would have their review the following year), or included in the denominator all children aged 2 or 3 who had a record of 2-2½ year review (including 3 year olds who had their review in the previous year (2017/18) and 2 year olds who were not due their review until the following year (2019/20)).

Equation 1: Estimation of the percentage of children with a 2-2½ year review

$$\text{Percentage of children with a 2-2.5 year review} = \frac{\text{Children aged 2 with a 2-2.5 year review}}{\text{(Children aged 2) - (Children aged 3 with a 2-2.5 year review)}}$$

Supplementary Material 3: Comparison of our results with experimental statistics from Public Health England: explanation of discrepancies

There are some slight discrepancies between our results and the experimental statistics published by PHE,(1) which report relative coverage of the 2-2½ year review in 2018/9 including by socioeconomic and demographic variables.

PHE has labelled its statistics as 'experimental', to be interpreted with caution and subject to further updates. This reflects the fact that PHE analyses were conducted rapidly, to support urgent policy needs with resources of 1 month of senior analyst time (personal communication with PHE, May 2021). Our analyses had a methodological focus, aiming to explore the completeness of CSDS data and how it might be best used for research. We used the 2-2½ year review as the substantive exemplar question for our foundational work on the quality and completeness of the CSDS data. Our study had 6 months of researcher time, supervisory support from two senior academic staff and input from the authorship team.

The summary below has been developed following conversations with colleagues at PHE

Discrepancies:

PHE report that children with a safeguarding vulnerability were slightly more likely to receive a 2-2½ year review than their peers were as we found the inverse.

PHE report that Looked after children were less likely to receive a 2-2½ year review than their peers which is consistent with our findings, but we found a greater difference than reported by PHE.

PHE report that children from ethnic minority groups were less likely to have their 2-2½ year review than children recorded as 'white'. In contrast we did not find any meaningful differences between the proportion of children in different ethnic groups who received their 2-2½ year review.

Probable explanation

1. local authorities included in analyses

Our research uses a research-ready subset of CSDS data from 33 local authorities with higher data completeness. For safeguarding vulnerabilities and Looked After Children analyses were restricted to 7 and 13 local authorities, respectively which had <10% missing data for these variables. PHE used data from all local authorities in England. AS part of our discussions with PHE, the analysts re-ran the anlayses on the 33 local authorities which we used, for comparison. Difference in sample is likely to explain the discrepancies in results for safeguarding vulnerabilities. When restricted to the 33 local authorities, PHE analyses also found that children with safeguarding vulnerabilities were slightly less likely to receive a review compared to their peers (OR 0.9). The difference in magnitude of results for Looked After Children (who were less likely to have a review in both sets of results) is likely due to differences in the local authorities included in the analyses. Our analyses might be less subject to misclassification of Looked After Children as analyses were restricted to local authorities with <10% missing data for this variable. There is also the possibility that the 13 local authorities with <10% missing data were systematically different from the 33 research ready local authorities and all local authorities in England.

The ethnic distribution of the research-ready dataset is different from the whole of CSDS, with the % of children in the mixed, Asian, Black and other categories lower in the research-ready dataset than in CSDS as a whole. This likely reflects regional differences in ethnicity. People in the Black, Asian, mixed and other ethnic groups are more likely to live in London than any other region in England, but only three London boroughs are in the high correlation subset. The PHE experimental statistics report substantial regional variation in the relative odds of receiving a 2-2½ year review for children in different ethnic groups. This means that any 'over-representation' of a region in the research-ready subset is likely to generate results substantially different from analyses of all local authorities in England. Alternatively, it might be that poor data quality in the entire CSDS compared to the research-ready subset has led to the difference in results.

2. Years of data and age of child

PHE used two years' worth of data and imputed data for children missing in one year who should appear in both. This imputation was not completed in our research, which only used data from 2018/9. PHE included data for aged 3, unlike our study, so may have picked up some 2-2.5 year reviews completed at age 3.

Note: the discrepancies in results for ethnicity are hardest to explain and persist even after PHE re-ran analyses on the 33 research ready local authorities used in the our analyses.

Conclusion:

Analyses of the CSDS data are sensitive to small changes in analytical approaches and sensitivity analyses should be carried out as routine.

References

1: Public Heath England, *Characteristics of children receiving Universal Health Visitor Reviews: Experimental analysis of the Community Services Dataset. Available at* https://www.gov.uk/government/publications/characteristics-of-children-receiving-universal-health-visitor-reviews *[last Accessed 25.02.21]*. 2021.
