# Supplementary material for: Variation in health visiting contacts for children in England: cross-sectional analysis of the 2–2½ year review using administrative data (Community Services Dataset, CSDS)
Source: BMJ Open. 2022 Feb 22;12(2):e053884. doi: 10.1136/bmjopen-2021-053884 (PMC8867374; doi:10.1136/bmjopen-2021-053884)
Supplement: Author's manuscript [file bmjopen-2021-053884.draft_revisions.pdf]

BMJ Open

BMJ Open is committed to open peer review. As part of this commitment we make the peer review history of every article we publish publicly available.

When an article is published we post the peer reviewers' comments and the authors' responses online. We also post the versions of the paper that were used during peer review. These are the versions that the peer review comments apply to.

The versions of the paper that follow are the versions that were submitted during the peer review process. They are not the versions of record or the final published versions. They should not be cited or distributed as the published version of this manuscript.

BMJ Open is an open access journal and the full, final, typeset and author-corrected version of record of the manuscript is available on our site with no access controls, subscription charges or pay-per-view fees (http://bmjopen.bmj.com).

If you have any questions on BMJ Open's open peer review process please email info.bmjopen@bmj.com

BMJ Open

Variation in health visiting contacts for children in England: cross-sectional analysis of the 2-2½ year review using administrative CSDS data

Journal:	*BMJ Open*
Manuscript ID	bmjopen-2021-053884
Article Type:	Original research
Date Submitted by the Author:	26-May-2021
Complete List of Authors:	Fraser, Caroline; UCL Great Ormond Street Institute of Child Health Population Policy and Practice<br>Harron, Katie; UCL Great Ormond Street Institute of Child Health Population Policy and Practice<br>Barlow, Jane; University of Oxford, Department of Social Policy and Intervention,<br>Bennett, Samantha; Kent Community Health NHS Foundation Trust<br>Woods, Geoffrey; East Sussex County Council, Early Help Data & Information Team<br>Shand, Jenny; UCL Partners, ; UCLPartners<br>Kendall, Sally; University of Kent,<br>Woodman, Jenny; UCL, Social Research Institute
Keywords:	PUBLIC HEALTH, Organisation of health services < HEALTH SERVICES ADMINISTRATION & MANAGEMENT, Child protection < PAEDIATRICS, Community child health < PAEDIATRICS

SCHOLARONE™
Manuscripts

I, the Submitting Author has the right to grant and does grant on behalf of all authors of the Work (as defined in the below author licence), an exclusive licence and/or a non-exclusive licence for contributions from authors who are: i) UK Crown employees; ii) where BMJ has agreed a CC-BY licence shall apply, and/or iii) in accordance with the terms applicable for US Federal Government officers or employees acting as part of their official duties; on a worldwide, perpetual, irrevocable, royalty-free basis to BMJ Publishing Group Ltd ("BMJ") its licensees and where the relevant Journal is co-owned by BMJ to the co-owners of the Journal, to publish the Work in this journal and any other BMJ products and to exploit all rights, as set out in our licence.

The Submitting Author accepts and understands that any supply made under these terms is made by BMJ to the Submitting Author unless you are acting as an employee on behalf of your employer or a postgraduate student of an affiliated institution which is paying any applicable article publishing charge ("APC") for Open Access articles. Where the Submitting Author wishes to make the Work available on an Open Access basis (and intends to pay the relevant APC), the terms of reuse of such Open Access shall be governed by a Creative Commons licence – details of these licences and which Creative Commons licence will apply to this Work are set out in our licence referred to above.

Other than as permitted in any relevant BMJ Author's Self Archiving Policies, I confirm this Work has not been accepted for publication elsewhere, is not being considered for publication elsewhere and does not duplicate material already published. I confirm all authors consent to publication of this Work and authorise the granting of this licence.

For peer review only

Title: Variation in health visiting contacts for children in England: cross-sectional analysis of the 2-2½ year review using administrative CSDS data

Authors

Dr Caroline Fraser, Research Fellow, UCL Great Ormond Street Institute of Child Health caroline.fraser@ucl.ac.uk

Dr Katie Harron, Associate Professor of Statistics, Population, Policy & Practice Dept, UCL Great Ormond Street Institute of Child Health. London. WC1N 1EH, UK k.harron@ucl.ac.uk

Professor Jane Barlow, Professor of Evidence Based Intervention and Policy Evaluation, Department of Social Policy and Intervention, University of Oxford OX1 2ER jane.barlow@spi.ox.ac.uk

Mrs Samantha Bennett, Consultant in Public Health, Kent Community Health NHS Foundation Trust, Trinity House, Eureka Park, Ashford TN25 4AZ samantha.bennett15@nhs.net

Mr Geoffrey Woods, Information Analyst, Early Help Data & Information Team, East Sussex County Council , Lewes, East Sussex, BN7 1UE geoffrey.woods@eastsussex.gov.uk

Dr Jenny Shand, Director, UCLPartners; Researcher, UCL; Care City Cohort Director, Care City W1T 7HA jenny.shand@uclpartners.com

Professor Sally Kendall, Professor of Community Nursing and Public Health, Centre for Health Services Studies, University of Kent, Canterbury, Kent, CT2 7NF s.kendall-608@kent.ac.uk

**Dr Jenny Woodman, Associate Professor of Child and Family Policy, UCL Social Research Institute, London, WC1H 00A j.woodman@ucl.ac.uk

**Corresponding author

Word count: 4101

Key words:

Health visitor; child; Health Services, Preventive; healthcare disparities

For peer review only

ABSTRACT

Objective

The 2-2½ year universal health visiting review in England is a key timepoint for assessing child development and offering support for children to reach school readiness by age 4 years. We aimed to establish which children do not receive their 2-2½ year review and whether they have additional (non-mandated) contacts with the health visiting team.

Design, setting, participants

Cross-sectional analysis of the 2-2½ year review and additional health visiting contacts for 181,130 children aged two in England in 33 local authorities in England in 2018/9, stratified by ethnicity, deprivation and 'vulnerability' indicators.

Analysis

We used a 'research-ready' subset of the Community Services Dataset (CSDS), administrative data capturing data on health visiting contacts for children in England. We calculated the percentage of children with a recorded 2-2½ year review and/or any additional health visiting contacts and average number of contacts, by child characteristic.

Results

Seventy-four percent of eligible children, 72% of those living in the most deprived areas, 83% with safeguarding vulnerability, and 44% of Looked After Children (LAC) had a 2-2½ year review recorded. Seventy-eight percent of eligible children, 78% of the most deprived, 80% of children with safeguarding vulnerability and 73% of LAC had any contact with the health visiting team recorded. Vulnerable and LAC children who saw the health visiting team in the year did so multiple times.

Conclusions

The health visiting service fell short of universal reach for children aged 2 years in England in 2018/9. Although the majority of vulnerable children in our sample saw a member of the health visiting team multiple times in the year, a substantial minority did have any recorded contact with the team. Further work is needed to establish the reasons for this, and potential solutions. There is an urgent need to improve the quality of national health visiting data.

For peer review only

ARTICLE SUMMARY: strengths and limitations of this study

- This is the first study to analyse the coverage and intensity of health visiting in England, also taking into account additional (non-mandated) contacts from the health visiting team.
- We addressed incompleteness in the national administrative data on health visiting in England (CSDS) by a) developing methods to identify a research-ready subset of the national data using comparisons to reference data sources b) limiting analyses to local areas with <10% missing data for vulnerability indicators. This approach minimises the likelihood that our results are an artefact of data quality, thereby increasing our confidence that they can be applied to future analyses of this and other national administrative datasets with variable completeness for local areas.
- However, our approach to dealing with incomplete data limits the generalisability of our results to the whole of England. This is particularly the case for our results about vulnerable children and different ethnic groups.
- We were reliant on the information recorded in the administrative data, all of which is entered by the health visiting teams. Although we restricted our analyses to local authorities with the most complete data, it is likely that there is still misclassification of children as vulnerable/not due to team members not being aware of everything that is happening in a child's life or professional concerns not meeting thresholds of certainty or severity for using the 'safeguarding vulnerability' code in the child's record.
- Despite the limitations, this is an important contribution to the evidence-base about how health visiting is delivered in England, which is foundational to making any improvements or modifications to the service.

For peer review only

INTRODUCTION

In England, there has been a sustained cross-government focus on identifying services and policies for babies and young children to "level up the opportunities for children, no matter where they come from or grow up" (Matt Hancock, July 2020).(1-5) These policies represent a response to the evidence that at age 5 years, certain groups of children are so far behind in terms of development that they will "struggle to ever catch up".(6) Children who start school with lower than expected levels of development are more likely to be excluded from school or have social services involvement by the time they are 11.(6) By age 16, disadvantaged children are 18 months behind their peers and 40% of this development gap had already emerged by the age of five.(6)

There is one universal intervention programme for preschool children in England, which specifically aims to address inequalities as well as promote health and development of young children: The Healthy Child Programme (HCP 0-5), led by Health Visitors (Fig 1). A key part of the HCP 0-5 is five mandated contacts by the health visiting team before a child turns 3 years old (Fig 1). The last of these reviews is at 2-2½ years, and represents a vital opportunity to assess a child's readiness to learn, their physical and social and emotional development and to identify any additional support needed to start school on a level footing with their peers.(6, 7) Since 2018, there has been particularly high cross-government policy focus on the role that the 2-2½ year review can play in reducing social inequalities in cognitive development.(8-10) As part of a holistic assessment of the child during the 2-2½ year review, practitioners are required to use the Ages and Stages Questionnaire (ASQ-3™ (11)), and since 2020, also the Early Language Identification Measure (ELIM). See Figure 1 for a more detailed explanation of these tools.

There is now extensive recognition that the critical 1001 days (conception to age 2) represents the best opportunity for intervening with the aim of levelling up and the Leadsom report (March 2021) highlights the need for every local authority to develop a Best Start for Life offer, in order to achieve this.(10) In order to evaluate the impact of specific policies and interventions delivered in the critical 1001 days, we need a measure of child development for all children at the end of this period (age 2) which is complete, accurate and available for analysis, such as the ASQ-3 and/or ELIM, which is collected as part of the universal 2-2½ year review. See Fig 1 for details of ASQ-3 and ELIM.

For peer review only

Despite recognition of the important role of the 2-2½ year review both for supporting individual families and for collecting data to support planning and policies at a local and national population level, there is evidence that a substantial proportion of children still do not receive a 2-2½ year review. Data from Public Health England (PHE) 'interim reporting metrics' ('metrics') indicate that 22% of eligible children in England did not have a record of 2-2½ year review in 2018/19 and in 2019/20, with substantial variation across the country (27-97%).(6, 12) The Children's Commissioner and PHE both estimate that 9 out of 10 children receiving a 2-2½ year review also had an ASQ-3 completed but other studies have found significant variability across England in the implementation and reporting of the ASQ-3.(13-15)

The sub-universal reach of the 2-2½ year review has prompted questions about whether some children are systematically more likely to miss out on a review. In 2020, the Children's Commissioner tried to ascertain if vulnerable children were differentially likely to have a 2-2½ year review than their peers but found that most local authorities did not collect the necessary data, concluding: “There is little evidence that local areas are ensuring that their vulnerable young children are checked”.(13) Since then, PHE has published experimental statistics (16), suggesting that the likelihood of receiving mandated reviews varies with ethnicity, and that children living in the most affluent areas of England were more likely to receive a review than children living in the more deprived areas.(10) However, these emerging social patterns of the mandated health visiting reviews, including the 2-2½ year review need further confirmation. Additionally, we do not know how patterns of the mandated reviews are similar to or differ from patterns of additional (non-mandated) contacts by the health visiting team. It may be, for example, that the children who miss out on a mandated review are seeing members of the health visiting team regularly for other reasons.

AIM

In this study, we used a national administrative dataset (the Community Services Dataset; CSDS (17)) to determine whether children from certain ethnic groups in England, or who had higher levels of deprivation or a safeguarding vulnerability were more likely to miss out on their 2-2½ year review in 2018/9 than their peers.

We aimed to quantify the proportion of children that had additional contacts from the health visiting team in 2018/9 (coverage), the frequency of these contacts (intensity) and how coverage and intensity of additional contacts varied according to ethnicity, deprivation and whether a child had safeguarding vulnerabilities recorded or was recorded as a Looked After Child.

For peer review only - http://bmjopen.bmj.com/site/about/guidelines.xhtml

METHODS

Study design and setting

This study comprises a cross-sectional analysis of coverage of the 2-2½ year health visiting review and coverage and intensity of additional health visiting contacts for children aged two in England in 2018/9, stratified by ethnic group, deprivation and 'vulnerability' indicators.

Data Source

The CSDS contains individual-level longitudinal administrative data from community services in England since 2015, including data on mandated and additional contacts with health visiting services. It is operated by NHS Digital, and providers of publicly-funded community services (including NHS trusts, private providers and the voluntary sector) are legally mandated to submit data. CSDS captures basic child characteristics (age, ethnicity), contacts with health visiting services (type, frequency, length, date) and a wider range of identified needs in children such as referral from or to specialist services. Quintiles of deprivation are derived from the Index of Multiple Deprivation, based on the child's postcode of usual address.

CSDS is the only child-level national source of information about health visiting in England but is a relatively new dataset, with outputs classified by NHS Digital as experimental. To our knowledge, the only published analysis of CSDS has been PHE's experimental statistics describing patterns in mandated reviews, cited by the Leadsom review. (10, 16)

Our 'research-ready' subset of data

We analysed a pseudonymised extract of CSDS that was held within PHE for the purpose of delivering PHE's core work programme and priorities, for contacts between 1 April 2018 and 31 March 2019. We checked the completeness of CSDS by comparing the number of eligible children and health visitor contacts recorded in CSDS with ONS data on births, health visitor contacts reported within PHE metrics, and anonymised health visiting data obtained directly from three LAs. To account for variation in the completeness of CSDS data across England and over time, we developed and applied methods for identifying a subset of 'research-ready' CSDS data for analysis (full details in Supplementary Material 1).

Our research-ready dataset for analyses of the 2-2½ year review in 2018/9 included 181,130 children from 33 local authorities. Children in the research-ready dataset were similarly deprived but less ethnically diverse than all children in England (see Supplementary Material 1). We identified a

For peer review only

further subset of 13 local authorities with 18,240 children with sufficiently complete data (<10% missing) for analyses of Looked After Child Status and 7 local authorities with 15,485 children with sufficiently complete data on the data item "Safeguarding Vulnerability".

Analyses

All analyses were conducted on the research-ready dataset. We estimated the percentage of eligible children who received a 2-2½ year review. Our denominator comprised all children aged 2 on 31 March 2019 minus the number of children who had the 2-2½-year review and were aged 3 on 31 March 2019 (see Supplementary Material 2 for full details). The numerator comprised children aged 2 on 31 March 2019 who had a 2-2½ year review scheduled and coded as 'attended' or with missing attendance data (5% of all 2-2½ year reviews).

We calculated the percentage of children who received any face-to-face contact with a member of the heath visiting team for any reason (including the mandated 2-2½ year review and additional contacts) in 2018/9, by location of contact (home/any other location). We calculated the median and interquartile range for the number of all attended contacts per child (including letters and phone calls) and face-to-face 'attended' contacts. We quantified the number of children with a recorded ASQ-3 but no 2-2½ year review recorded, as an indication of 2-2½ year reviews that took place but might not have a 2-2½ year review code attached.

We stratified results by ethnicity, deprivation (IMD quintile) and vulnerability, where a 'vulnerable' child was defined as one with a code indicating a safeguarding vulnerability or Looked After Child at any point during 2018/19. Both codes are entered manually by a member of the health visiting team as part of their usual care of the child. The 'safeguarding vulnerability' code indicates factors such as referral from police or children's social care, significant injury, known or suspected domestic abuse, worrying parent behaviour or concerns about parental mental health. (18) Analysis of these characteristics was only conducted for LAs with less than 10% missing data for these variables (N=13 for Looked After Children and N=7 for safeguarding vulnerability). As IMD was complete for all 33 LAs and ethnicity had a similar level of completeness (16-23% missing) across all LAs, we included 33 LAs in analyses of these variables.

Patient and Public Involvement

We did not conduct any patient or public involvement for this study.

RESULTS

For peer review only - http://bmjopen.bmj.com/site/about/guidelines.xhtml

In our research-ready dataset, 74% of eligible children received their 2-2½ year review, 76% had any face-to-face contact with the health visiting team in the previous 12 months (including a 2-2½ year review and additional contacts) and 78% had *any* contact, including letters or phone calls (Table 1). If we assume that **all** the children with an ASQ-3 record had a 2-2½ year review, our estimate of children with a 2-2½ year review increases to 81%.

More deprived children were slightly more likely to receive a 2-2½ year review than less deprived children but the same pattern was not evident for additional contacts (Table 1). The proportion of eligible children with a 2-2½ year review did not vary meaningfully according to whether the child had a Safeguarding Vulnerability recorded or by ethnicity (see confidence intervals, Table 1). Looked After Children were much less likely to have a 2-2½ year review recorded compared to other children (44% compared to 69%, Table 1). However, this difference disappeared when all face-to-face additional contacts with the health visiting team were included. The lower proportion of 2-2½ year reviews for Looked After Children was not explained by missed appointments: all 90 Looked After Children with scheduled 2-2½ year reviews were recorded as 'attended'.

The majority of all children (76%), children with safeguarding vulnerabilities recorded (78%) and Looked After children (71%), had a record of a contact with health visiting services, either for a 2-2½ year review or as an additional contact (Table 1). The most common face-to-face activities (n=110,780) for children aged 2, other than the 2-2½ year review, were “other” (31%, n=34,180), assessments (23%, n=26,015), counselling, advice or support (17%, n=18,875) and clinical interventions (15%, n=17,145), which include individualised care plans, for example parenting advice, support with behaviour and child development. These figures include multiple activities recorded for the same child (including on the same day).

A substantial proportion of all children (24%), children with a ‘safeguarding vulnerability’ recorded (22%) and Looked After children (29%) did not have a record of either a 2-2½ year review or any other face-to-face contact (Table 2). Most of the children without a face-to-face contact did not have a record of letters or telephone calls from health visiting services, suggesting no contacts were attempted. On average, children that had any type of contact with health visiting services saw a member of the team once in this period, compared to 3 times for children with a safeguarding vulnerability code and 5 times for children with a Looked After Child code (Table 2).

For peer review only - http://bmjopen.bmj.com/site/about/guidelines.xhtml

Table 1: Percentage of children aged 2 with a 2-2½ year review or any contact (face-to-face or other medium) with a health visiting team

Child characteristic	With a 2-2½ year review attended		Children aged 2 on 31 March 2019 with any contact with health visiting teams			
			Face-to-face contacts		Any (including letters and telephone)	
	% of eligible* children (95% CI)	Number with a review / eligible children	% of eligible* children (95% CI)	Number with a review / eligible children	% of eligible* children (95% CI)	Number with a review / eligible children
**All children (33 LAs)**	74% (73%, 74%)	55,975 / 75,960	76% (75%, 76%)	70,695 / 93,525	78% (78%, 78%)	73,065 / 93,525
**Safeguarding vulnerability code (7 LAs)**						
No	86% (85%, 86%)	13,090 / 15,280	80% (80%, 81%)	14,550 / 18,150	83% (83%, 84%)	15,120 / 18,150
Yes	83% (78%, 88%)	170 / 205	78% (73%, 83%)	195 / 250	80% (75%, 85%)	200 / 250
**Looked After child (13 LAs)**						
No	69% (69%, 70%)	19,475 / 28,035	77% (77%, 78%)	26,820 / 34,705	79% (78%, 79%)	27,355 / 34,705
Yes	44% (37%, 51%)	90 / 205	71% (65%, 77%)	170 / 240	73% (67%, 79%)	175 / 240
**Quintile of IMD (33 LAs)**						
1 (most deprived)	72% (72%, 73%)	14,295 / 19,800	78% (78%, 79%)	18,620 / 23,785	81% (80%, 82%)	19,305 / 23,785
2	72% (71%, 72%)	11,065 / 15,475	76% (76%, 77%)	14,420 / 18,910	79% (78%, 79%)	14,870 / 18,910
3	73% (72%, 74%)	10,685 / 14,680	74% (73%, 74%)	13,630 / 18,465	76% (75%, 77%)	14,035 / 18,465
4	75% (75%, 76%)	9,850 / 13,075	73% (73%, 74%)	11,985 / 16,345	76% (75%, 77%)	12,420 / 16,345
5 (least deprived)	78% (77%, 78%)	10,045 / 12,940	76% (75%, 76%)	12,165 / 16,015	78% (78%, 79%)	12,565 / 16,015
**Ethnicity (33 LAs)**						
White	73% (72%, 73%)	33,455 / 45,920	73% (72%, 73%)	42,360 / 58,150	75% (75%, 75%)	43,655 / 58,150
Mixed	79% (78%, 80%)	5,875 / 7,405	90% (89%, 91%)	7,390 / 8,210	91% (91%, 92%)	7,505 / 8,210
Asian or Asian British	76% (75%, 78%)	2,920 / 3,825	80% (78%, 81%)	3,555 / 4,470	81% (80%, 82%)	3,620 / 4,470
Black or Black British	73% (71%, 75%)	1,185 / 1,620	80% (79%, 82%)	1,530 / 1,905	82% (80%, 83%)	1,555 / 1,905
Other	63% (61%, 66%)	1,045 / 1,650	71% (69%, 73%)	1,450 / 2,030	75% (73%, 77%)	1,520 / 2,030

LAs: Local Authorities. IMD: Index of Multiple Deprivation. *Note: The percentage of children aged 2 with any face-to-face contact may be lower than the percentage of children with a 2-2½ year review because we reduced the number of children in the denominator for the 2-2½ year review to account for children with the 2-2½ year review aged 3 (based on age at end of 2018/19), and a small number of 2-2½ year reviews were not be face-to-face (see Supplementary Material 2 for full explanation).

For peer review only

Table 2: Median contacts per child for children aged 2 with any recorded contact during 2018/19

**Child characteristic**	**Median (interquartile range) number of contacts aged 2 for children with contacts**	
	**Any contact**	**Face-to-face**
**All children (33 LAs)**	1 (1 to 2)	1 (1 to 2)
**Safeguarding factors (7 LAs)**		
No	1 (1 to 2)	1 (1 to 2)
Yes	3 (2 to 7)	3 (1 to 7)
**Looked After child (13 LAs)**		
No	1 (1 to 2)	1 (1 to 2)
Yes	6 (3 to 10)	5 (3 to 9)
**Quintile of IMD (33 LAs)**		
1 (most deprived)	2 (1 to 4)	2 (1 to 3)
2	2 (1 to 3)	1 (1 to 3)
3	1 (1 to 3)	1 (1 to 2)
4	1 (1 to 2)	1 (1 to 2)
5 (least deprived)	1 (1 to 2)	1 (1 to 2)
**Ethnicity (33 LAs)**		
White	1 (1 to 3)	1 (1 to 2)
Mixed	1 (1 to 3)	1 (1 to 2)
Asian or Asian British	2 (1 to 2)	1 (1 to 3)
Black or Black British	2 (1 to 4)	1 (1 to 3)
Other	1 (1 to 3)	1 (1 to 2)

LAs: Local Authorities. IMD: Index of Multiple Deprivation.

For peer review only

Overall, 28% of children with a face-to-face contact were seen at home, and the percentage of at home contacts was greater for Looked After children (63%), children with safeguarding vulnerabilities (78%), and children living in the most deprived areas (38%) (Table 3). However, if all contacts with missing data on location (12%; 8805/70,695) were in fact home visits, the proportion of all face-to-face contacts that were in a child's home could be as high as 40%.

For peer review only

For peer review only - http://bmjopen.bmj.com/site/about/guidelines.xhtml

Table 3: Percentage of children aged 2 with a record of a face-to-face contact in any location and at home by child characteristic

Child characteristic	Children aged 2 with a record of a face-to-face contact [1]			
	In any location		At home	
	% (95% CI)	Children with a contact / children aged 2	% (95% CI)	Children with a contact / children aged 2
**All children (33 LAs)**	76% (75%, 76%)	70,695 / 93,525	28% (27%, 28%)	26,130 / 93,525
**Quintile of IMD (33 LAs)**				
1 (most deprived)	78% (78%, 79%)	18,620 / 23,785	38% (37%, 38%)	8,920 / 23,785
2	76% (76%, 77%)	14,420 / 18,910	28% (27%, 29%)	5,345 / 18,910
3	74% (73%, 74%)	13,630 / 18,465	27% (26%, 27%)	4,900 / 18,465
4	73% (73%, 74%)	11,985 / 16,345	24% (23%, 24%)	3,845 / 16,345
5 (least deprived)	76% (75%, 76%)	12,165 / 16,015	20% (19%, 21%)	3,210 / 16,015
**Ethnicity (33 LAs)**				
White	73% (72%, 73%)	42,360 / 58,150	27% (27%, 28%)	15,985 / 58,150
Mixed	90% (89%, 91%)	7,390 / 8,210	25% (24%, 26%)	2,020 / 8,210
Asian or Asian British	80% (78%, 81%)	3,555 / 4,470	32% (31%, 33%)	1,435 / 4,470
Black or Black British	80% (79%, 82%)	1,530 / 1,905	32% (31%, 33%)	595 / 1,905
Other	71% (69%, 73%)	1,450 / 2,030	31% (29%, 33%)	630 / 2,030
**Looked After children (13 LAs)**				
No	77% (77%, 78%)	26,820 / 34,705	27% (27%, 28%)	9,500 / 34,705
Yes	71% (65%, 77%)	170 / 240	63% (56%, 67%)	150 / 240
**Safeguarding factors (7 LAs)**				
No	80% (80%, 81%)	14,550 / 18,150	38% (38%, 39%)	6,420 / 18,150
Yes	78% (73%, 83%)	195 / 250	65% (59%, 70%)	130 / 205

[1] Rounded to nearest 5 to reduce risk of disclosure as data is subnational; LAs: Local Authorities; IMD: Index of Multiple Deprivation

For peer review only

DISCUSSION

Main findings

The majority (>70%) of eligible children in our sample of 33 local authorities, including those defined as vulnerable, were engaged with health visiting services and received their 2-2½ year review. Our results indicate that children in the most deprived areas were *slightly* more likely to miss out on a 2-2½ year review and Looked After children were *much* more likely to miss out on this review. However, when all additional contacts were included, the pattern was reversed (for deprivation) or disappeared (for Looked After children). We also found that Looked After and the most deprived children received more intensive health visiting than their peers, with more frequent face-to-face contacts in the 12 month period. Although we found no evidence that children with a safeguarding vulnerability recorded were any more or less likely to receive a 2-2½ year review or an additional contact than their peers, we did find that when this group had at least one face-to-face contact, they received more intensive health visiting than average.

Our results suggest that there are groups of high need children (e.g. deprived children and Looked After children) who are engaged with the health visiting team but do not receive their mandated 2-2½ year review. This raises questions about whether the 2-2½ year review and/or the ASQ-3 is perceived as suitable for high need groups in the population. It also highlights the importance of including additional contacts in analyses when investigating whether the delivery of health visiting in England is socially patterned.

Our results also suggest that there might be two distinct groups of vulnerable children with regard to the receipt of health visiting. We identified a majority group of vulnerable children who received multiple face-to-face contacts within a 12-month period, often in the child's home. This group appears to be receiving intensive health visiting, consistent with the model of proportionate universalism that underpins health visiting in England (a universal service for all children but with greater support and service provision for children in families with identified needs). The second group comprised a substantial minority of vulnerable children who were not in contact with the health visitor team at all: 22% of children with safeguarding vulnerabilities and 29% of Looked After children did not have a record of either a 2-2½ year review or any other face-to-face additional contact in the year. As we did not find evidence of attempted contacts with these children in CSDS, there is a real possibility that these vulnerable children might not have heard from or seen a member of the health visiting team within the year.

For peer review only - http://bmjopen.bmj.com/site/about/guidelines.xhtml

Our results on the 2-2½ year review are largely consistent with the experimental statistics published by PHE, with the exception of ethnicity.(16) PHE found that children from ethnic minority groups were less likely than white children to receive the mandated reviews, including the 2-2½ year review.(16) The difference is likely due to poor data completeness in CSDS and different analytical approaches to dealing with this incompleteness, see supplementary material 3 for a detailed explanation.

Implications

Our data indicates that the health visiting service fell short of universal reach for children aged 2 years in England in 2018/9. Together, our results and the PHE experimental statistics suggest that 2-2½ year reviews are socially patterned. However, the scale of the difference and whether it exists for different ethnic groups remains uncertain, largely as a result of poor data completeness in CSDS (supplementary material 3). Poor data remains a significant barrier to understanding how health visiting is currently delivered, and currently the only available national data for England can only generate results which should be treated as hypotheses, with a need for further exploration.

Although the health visiting service seems to be achieving a model of proportionate universalism for **some** children with known vulnerabilities (with multiple visits a year, often in the child's home), there was also a substantial minority of vulnerable children without any recorded contact from the health visiting team. This is of significant concern, given the likely concentration of health and welfare need in these groups and makes it unlikely that the 2-2½ year review is helping to address inequalities in school readiness as intended. The secondary effects of the COVID-19 pandemic, including heightened need and reduced workforce capacity, are likely to exacerbate the shortfall both in universal reach and provision of extra intensive services where needed.(19)

Conclusions

Since completion of this study, the HCP is being revised to be 'universal in reach and personalised in response'.(20) Our study evidences the gap between this policy ambition and the current reality of health visiting, based on children aged two in 33 local areas in 2018/9.(21) Further work is needed to establish reasons for low coverage of the 2-2½ year review in Children Looked After and to generate recommendations about the most suitable way of delivering the 2-2½ year review to Looked After Children and other groups with known complex needs or vulnerabilities.

In order to produce reliable estimates, researchers require access to high quality national data from health visiting services linked to other datasets (such as health, social care and education) for the

For peer review only - http://bmjopen.bmj.com/site/about/guidelines.xhtml

whole of England.(22) CSDS is currently very incomplete: we could only use data from 1 in 5 local authorities in England in our analysis. (23) NHS Digital and PHE are undertaking quality improvement work to address the missing data in CSDS. The findings of this study suggest that policies and/or funding to accelerate the pace of CSDS quality improvements should be considered at a national level. Further work is needed both on how best to support local practitioners to improve data recording in their systems and on how data transfer from local systems into CSDS might be improved, given the evidence that some data on health visiting contacts in local systems does not make it into CSDS.(23)

Limitations of this study

Due to our extract of CSDS, we could not follow a child over time, which might mean we slightly underestimate 2-2½ year reviews as some children may have had their 2-2½ year review just before or after our study period. As administrative data, CSDS codes will not capture everything health visitors identify or deliver. Information might be coded with high accuracy on local systems but 'lost' in the transfer to NHS Digital, e.g. with use of slightly different codes. There is some evidence of this, based on our comparison of two local areas with CSDS data.(23) Second, contacts may be miscoded. Although our analyses of ASQ-3 records provide some evidence of this, the consistency of our results with other data(23) suggests that miscoding does not explain the 1 in 5 children 'missing' a 2-2½ year review. We know that much abuse and neglect of children will not be coded in administrative data, even if it is suspected by professionals(24, 25) which will lead to under-estimating differences between vulnerable and other children. Many data items were too poorly completed to use, for example child disability and staff type (e.g. health visitor, nursery nurse). CSDS does not capture quality or meaning of the interaction between health visitors and parents. To obtain a full picture of health visiting activity in England, and the contribution of this service to child health and wellbeing, all types of LAs need to be investigated using a triangulation of data sources, including CSDS, locally held data, surveys and in-depth qualitative data collected from professionals and families, as a study in Scotland is attempting.(26)

Our results cannot be confidently generalised to all children in England. The children in the 'research-ready' data were not nationally representative in terms of ethnicity (Supplementary Material 1) and the excluded LAs might be systematically different from those we included which had more complete data. Although our findings should be treated as hypotheses, this study marks an important first step in making use of existing, routinely collected data to understand the coverage and intensity of health visiting services in England.

For peer review only

For peer review only - http://bmjopen.bmj.com/site/about/guidelines.xhtml

FUNDING

This study/project was supported by the National Institute for Health Research (NIHR) Policy Research Programme (PR-PRU-1217-21301). The views expressed are those of the author(s) and not necessarily those of the NIHR, the Department of Health and Social Care or Public Health England.

This project is delivered as part of PHE's 2020/21 Life Course Intelligence business plan for child and maternal health in order to understand CSDS data quality and its suitability for further analysis to understand trends, variation and inequalities in health visiting and outcomes for children in the early years. An honorary contract with PHE enabled the researcher to work as part of the analytical team and analyse the CSDS for this purpose.

This research was supported in part by the NIHR Great Ormond Street Hospital Biomedical Research Centre and the Health Data Research UK (grant No. LOND1), which is funded by the UK Medical Research Council and eight other funders. KH is supported by funding from NIHR (17/99/19).

AUTHOR CONTRIBUTIONS

JW and KH designed and supervised analyses. CF carried out all analyses. SB, GW and JS provided access to locally held data and gave expert advice on data. All authors contributed to the interpretation of results and commented on multiple versions of the paper, written by JW and CF.

We would like to thank individuals in Public Health England for their comments and advice, including Wendy Nicholson within Nursing, Maternity and Early years and Kate Thurland and Helen Smith. We thank Professor Ruth Gilbert at UCL Great Ormond Street Institute of Child Health for input into study design, methods and drafts. We are grateful to Care City, Barking and Dagenham, Havering and Redbridge CCG, London Borough of Barking and Dagenham, East Sussex County Council and East Sussex Healthcare Trust and Kent County Council and Community Health NHS Foundation Trust for access to their local data and knowledge and expertise. We thank individuals at the Institute of Health Visiting for comments on drafts of the report(23) that preceded this paper.

None of the authors have any competing interests to declare.

DATA AVAILABILITY

The authors do not have permission for onward sharing of the individual-level data underlying this article. Requests to access the CSDS data can be made to NHS Digital. The study was approved by UCL Institute of Education Research Ethics Committee on 01.05.20 (Ref 1333).

For peer review only - http://bmjopen.bmj.com/site/about/guidelines.xhtml

Figure Legends

Figure 1: Delivery of the Health Child Programme (HCP) by Health Visitors and use of ASQ-3 and ELIM in the 2-2½ year review

For peer review only

For peer review only - http://bmjopen.bmj.com/site/about/guidelines.xhtml

References

1. Gov.uk/news. New focus on babies' and children's health as review launches. 2020. Available at https://www.gov.uk/government/news/new-focus-on-babies-and-childrens-health-as-review-launches [last accessed 28.09.20]
2. Marmot M, Goldblatt P, Allen J. Fairer society, healthy lives (the Marmot review). 2010. Available at http://www.instituteofhealthequity.org/resources-reports/fair-society-healthy-lives-the-marmot-review/fair-society-healthy-lives-full-report-pdf.pdf [last accessed 29.09.20].
3. A cross-party manifesto. The 1001 Critical Days: the importance of the conception to age 2 period. 2014. Available at https://www.wavetrust.org/1001-critical-days-the-importance-of-the-conception-to-age-two-period [last accessed 29.09.20].
4. Early Years and Family Support Ministerial Group. First 1000 Days of Life. 2018.
5. National Health Service. The NHS Long Term Plan. 2019. Available at https://www.longtermplan.nhs.uk/wp-content/uploads/2019/08/nhs-long-term-plan-version-1.2.pdf [last accessed 29.09.20].
6. Office for the Children's Commissioner. Best Beginnings in the Early Years: A proposal for a new early years guarantee to give all children in England the best start in life. 2020. Available at https://www.childrenscommissioner.gov.uk/wp-content/uploads/2020/07/cco-best-beginnings-in-the-early-years.pdf.[Last Accessed 25.02.21]
7. Department for Health. Universal Health Visitor Reviews: advice for local authorities in delivery of the mandated universal health visitor reviews from 1 October 2015. Available at https://www.gov.uk/government/publications/universal-health-visitor-reviews-advice-for-local-authorities [Last Accessed 25.02.21]
8. Public Health England. Early language identification measure and intervention. Available at https://assets.publishing.service.gov.uk/government/uploads/system/uploads/attachment_data/file/939872/ELIM_Handbook_December-2020.pdf [Last accessed 09.02.21]. 2020.
9. Public Health England, Department of Health and Social Care. Early language identification measure and intervention: guidance handbook. 2020. Available at https://assets.publishing.service.gov.uk/government/uploads/system/uploads/attachment_data/file/939872/ELIM_Handbook_December-2020.pdf [last accessed 10.05.21].
10. HM Government. The Best Start for Life: a vision for the 1,001 Critical Days. 2021. The Early Years Healthy Development Review Report. Available at https://assets.publishing.service.gov.uk/government/uploads/system/uploads/attachment_data/file/973112/The_best_start_for_life_a_vision_for_the_1_001_critical_days.pdf [last accessed 10.05.21]
11. Squires J, Brickers D, Twombley E, Nickel R, Clifford J, Murphy K. Ages & Stages Questionnaires®, Third Edition (ASQ-3™): A Parent -Completed Child Monitoring System. Baltimore: Paul H. Brookes Publishing Co., Inc.; 2009.
12. Public Health England. Health visitor service delivery metrics: 2018 to 2019 2019.
13. Office for the Children's Commissioner. Best beginnings in the early years: Technical report two. Available at https://www.childrenscommissioner.gov.uk/wp-content/uploads/2020/07/cco-best-beginnings-in-the-early-years-tech-report-2.pdf [last accessed 10.02.21]. 2020.
14. Kendall S, Nasg A, Braun A, Bastug G, Rougeux E, Bedford H. Evaluating the use of a population measure of child development in the Healthy Child Programme Two Year Review. Available at https://kar.kent.ac.uk/54584/1/evaluating-the-use-of-a-population-measure-of-child-development-in-the-healthy-child-two-year-review.pdf [last accessed 09.02.21]. 2014.
15. Kendall S, Nash A, Braun A, Bastug G, Rougeaux E, Bedford H. Acceptability and understanding of the Ages & Stages Questionnaires(R), Third Edition, as part of the Healthy Child Programme 2-year health and development review in England: Parent and professional perspectives. Child Care Health Dev. 2019;45(2):251-6.
16. Public Heath England. Characteristics of children receiving Universal Health Visitor Reviews: Experimental analysis of the Community Services Dataset. Available at

For peer review only - http://bmjopen.bmj.com/site/about/guidelines.xhtml

https://www.gov.uk/government/publications/characteristics-of-children-receiving-universal-health-visitor-reviews [last Accessed 25.02.21]. 2021.
17. NHS Digital. Community Services Data Set: NHS Digital; 2020 [updated 16/04/2020. Available from: https://digital.nhs.uk/data-and-information/data-collections-and-data-sets/data-sets/community-services-data-set. [Last accessed 26.05.21]
18. NHS Digital. Community Services Data Set technical output specification. 2018. [Available from: https://digital.nhs.uk/data-and-information/data-collections-and-data-sets/data-sets/community-services-data-set/community-services-data-set-technical-output-specification. [Last accessed 26.05.21]
19. Barlow J, Bach-Mortensen A, Homonchuk O, Woodman J. The impact of the COVID-19 pandemic on services from pregnancy through age 5 years for families who are high risk of poor outcomes or who have complex social needs. Available at https://www.ucl.ac.uk/children-policy-research/sites/children-policy-research/files/the_impact_of_the_covid-19_pandemic_on_services_from_pregnancy_through_age_5_years_final_report_101120.pdf [Last accessed 11.02.21]. 2020.
20. Department of Health and Social Care. Advancing our health: prevention in the 2020s (consultation). 2019. Available at www.gov.uk/government/consultations/advancing-our-health-prevention-in-the-2020s [last accessed 11.12.19].
21. Bryar RM, Cowley DS, Adams CM, Kendall S, Mathers N. Health visiting in primary care in England: a crisis waiting to happen? *Br J Gen Pract.* 2017;67(656):102-3. Available https://www.ncbi.nlm.nih.gov/pmc/articles/PMC5325624/ [Last accessed 25.05.21]
22. ADRUK webpage. ECHILD: Linking children's health and education data for England. 2021. Available at https://www.adruk.org/our-work/browse-all-projects/echild-linking-childrens-health-and-education-data-for-england-142/ [Last accessed 11.02.21]
23. Fraser C, Harron K, Barlow J, Bennett S, Woods G, Shand J, et al. 2020. How can we use the community services dataset (CSDS) for research into health visiting? Available at https://www.ucl.ac.uk/children-policy-research/sites/children-policy-research/files/using_csds_for_research_report_08.10.20.pdf [last accessed 14.01.21].
24. Woodman J, Allister J, Rafi I, de Lusignan S, Belsey J, Petersen I, et al. A simple approach to improve recording of concerns about childmaltreatment in primary care records: developing a quality improvement intervention. Br J Gen Pract. 2012;62(600):e478-e86. Available at https://bjgp.org/content/62/600/e478 [Last accessed 25.05.21]
25. Woodman J, Hodson D, Gardner R, Cuthbert C, Woolley A, Allister J, et al. The GP's role in responding to child maltreatment. London, NSPCC 2014. Available at https://www.ehcap.co.uk/content/sites/ehcap/uploads/NewsDocuments/219/RCGP-GP-Role-responding-to-child-maltreatment-July-2014-ashx.PDF [Last accessed 25.05.21]
26. Doi L, Morrison K, Astbury R, Eunson J, Horne MA, Jepson R, et al. Study protocol: a mixed-methods realist evaluation of the Universal Health Visiting Pathway in Scotland. BMJ open. 2020;10(12):e042305.Available at https://bmjopen.bmj.com/content/10/12/e042305 [Last accessed 25.05.21]
27. Public Health England. Health Visiting and school nursing service delivery model. London, PHE 2021. Available at https://www.gov.uk/government/publications/commissioning-of-public-health-services-for-children/health-visiting-and-school-nursing-service-delivery-model [Last accessed 25.05.21]
28. Department for Health. Healthy Child Programme. Pregnancy and the First Five Years. London, PHE 2009.
29. Institute of Health Visiting. Health visting in England: a vision for the future. London, iHV 2019. Available at https://ihv.org.uk/our-work/our-vision/ [Last accessed 25.05.21]
30. Department of Health. Universal Health Visitor Reviews: advice for local authorities in delivery of the mandated universal health visitor reviews from 1 October 2015. London, DH 2015.

For peer review only - http://bmjopen.bmj.com/site/about/guidelines.xhtml

31. Public Health England. Best start in life and beyond: Improving public health outcomes for children, young people and families. Guidance to support the commissioning of the Healthy Child Programme 0-19: Health Visiting and school nursing services. Commissioning guide 2. London, PHE 2018. Available at https://assets.publishing.service.gov.uk/government/uploads/system/uploads/attachment_data/file/969168/Commissioning_guide_1.pdf [Last accessed 25.05.21]
32. Squires J, Bricker D, Twombley E. Ages & stages questionnaires: Social-emotional (Vol. 2). Baltimore, Paul H. Brookes Publishing Company 2002.
33. Public Health England. Identifying and supporting children's early language needs. Summary report. 2020. Available at https://assets.publishing.service.gov.uk/government/uploads/system/uploads/attachment_data/file/939833/ELIM_Summary_report_December-2020.pdf [Last accessed 26.05.21]. 2020.
34. Department for Education. Unlocking talent, fulfilling potential. A Plan for improving social mobility in England. 2017. Available at https://assets.publishing.service.gov.uk/government/uploads/system/uploads/attachment_data/file/667690/Social_Mobility_Action_Plan_-_for_printing.pdf [last accessed 26.05.21].

For peer review only

Fig 1

Delivery of the Health Child Programme (HCP) by Health Visitors

The Healthy Child Programme (HCP) is designed to promote the health and development of young children, and prevent and mitigate the impact of adversity and inequalities in early childhood.(27, 28). The HCP is delivered by health visitors: registered nurses or midwives who have undertaken an additional Specialist Community Public Health Nursing qualification. As part of the HCP, LAs are required to commission delivery of a minimum of five contacts for every child and family in England before the child is aged 5 years in which health visitors or other members of the team such as staff nurses or nursery nurses review the child's health and development in the context of family health and environment, offer support in a range of areas, and signpost to community resources such as children's centres, child care and wider early years services.(29) Health visiting contacts can be delivered through home visits, individual or group clinic appointments, or phone calls and are supported by administrative activity such as letters to families.(30) The five mandated reviews, also known as 'universal health reviews', are an antenatal visit after 28 weeks of pregnancy, a new birth visit within 14 days, a 6 to 8 week review, an age one year review and a 2-2½ year review.(27)

Ages and Stage Questionnaire (ASQ-3)

The ASQ-3 is a tool to measure child development. Policy guidance for England states that the ASQ-3 should always be used as part of the 2-2½ year review in order that the ASQ-3 domain scores can be collected and used as population measure of child development, including at a local level to measure and then put in place interventions to address inequalities in child development "between lowest and highest IMD areas".(31) Practitioners might also use the ASQ-SE™, social and emotional questionnaire.(32) A study in 4 sites in England (2014) found confusion among parents and health care professionals about the purpose of ASQ-3, namely whether it was a tool for assessing a child's individual development or a population measure of child development.(14) This study recommends ASQ-3 be used as as one tool in the wider assessment of a child's health and development.(14)

Early Language Identification Measure (ELIM)

ELIM-E is a tool designed for use in the 2-2½ year review in order to identify children with speech, communication or learning needs and which should be used as part of a local pathway to identify and support individual children towards school readiness and, ultimately, reduce inequalities in school readiness across the population.(8, 33) The Early Language Identification Measure (ELIM) and accompanying intervention was commissioned in 2018 as part of the UK government's Social Mobility Action Plan,(34) with guidance issued to commissioners, service leads and practitioners in late 2020.(9, 33) Unlike the ASQ-3 tool, Public Health England and Department of Health and Social Care recommend the use of ELIM-E for assessing need in individual children.

For peer review only

For peer review only

Supplementary Material 1: Identifying a 'research-ready' subset of CSDS for use in research into the 2-2½ year review

The material in supplementary material 1 and 2 is based on our report: https://www.ucl.ac.uk/children-policy-research/sites/children-policy-research/files/using_csds_for_research_report_08.10.20.pdf

Methods

Identifying "high correlation" data for local authorities

To identify local authorities within CSDS with sufficiently complete data for research into 2-2½ year reviews, we created two indicators of how well data was recorded in CSDS and sense-checked these indicators with a Consultant in Public Health working with a Health Visiting Service (SB), an information analyst who manages health visiting data (GW) and the lead for PHE metrics (KT). The indicators measure the agreement between CSDS and aggregate reference data (PHE metrics or ONS births). Based on these indicators, we categorised local authorities as having high, moderate or low correlation between data in CSDS and reference data.

The indicators are based on the number of eligible children and children who had 2-2½ year reviews recorded (Table A1). These indicators were selected to account for the two mechanisms of under-recording in CSDS that would affect research into health visiting activity. First, some children may be missing completely from the data. If children are missing from CSDS, we do not have accurate data on how many children are eligible for health visiting and therefore do not have an accurate denominator to calculate the percentage of children who received contacts. We were able to determine whether the number of eligible children were accurate through comparisons between CSDS and ONS birth statistics. We focused on "eligible children" who were aged 0 because we did not have reliable reference data on the number of children aged 2 or 3 to compare with CSDS. Second, contacts with health visitors may be under-recorded. We were able to determine whether the number of contacts were accurate through comparisons between CSDS and PHE metrics.

Table A1: The indicators and limits for identifying high or moderate correlation local authorities in CSDS

Indicator	Limits	
	High correlation	Moderate correlation
**(i) Eligible children:** Agreement between the number of children aged 0 at the end of the reporting year in CSDS and ONS birth statistics	- 10% to +20%	-30% to +30%
**(ii) 2-2.5 year reviews:** Agreement between the number of children with a 2-2.5 year review in CSDS and PHE metrics	-15% to +15%	-40% to +40%

We used local data from East Sussex and Kent to select appropriate limits for the high and moderate correlation categories, by comparing local data to the reference data. The percentage difference between the local data and PHE metrics ranged from -10% to +1% for the new birth visit and 2-2½ year review (See Table A1). Based on this, we considered a difference between CSDS and PHE

For peer review only

metrics of +/-15% to be appropriate for high correlation and +/-40% appropriate for moderate correlation. We expect the true number of "eligible children" (children born in 2018/19) to be higher than the number of children born in England, because CSDS should include children born elsewhere who moved to England before their first birthday. This was true in our comparisons of local data and ONS births, where there were 3-8% more children aged <1 year in the local data compared to ONS births. This is reflected in the limits we selected for high correlation for eligible children (-10% to +20%).

Local authorities that did not meet either high or moderate criteria were classified as low correlation. As for many local authorities agreement with the indicators varied by quarter, we allowed local authorities to move between categories by quarter. This means that data from specific time periods within a local authority could be considered as high correlation and therefore included in analyses. The contact date was used to allocate children who had a contact to a local authority quarter (and therefore to a correlation category). For children without contacts (i.e. to calculate the denominator population), we could not determine which child became eligible in each quarter. Therefore, we calculated the total number of children per local authority divided by four to estimate the number of children per quarter.

Are children in the high correlation local authorities nationally representative?

To draw conclusions from research using the subset of children in the high correlation group we must understand whether results can be extrapolated to children in other local authorities in CSDS, and to all children whether or not they were recorded in CSDS. To determine whether the subset of local authorities were representative, we focused on the IMD quintile and ethnicity because of the completeness of these variables in CSDS (IMD is complete for all children in the study and ethnicity is complete for 82% of children) and because there were a substantial number of children within each IMD or ethnicity category.

To determine whether the chances of a child being recorded in CSDS varied by IMD quintile and ethnicity, we compared the distribution of IMD and ethnicity in ONS births to children in CSDS who were born in 2018/19. We also compared the distribution of IMD quintile and ethnicity of children aged <5 years in CSDS and local data for two local authorities (East Sussex and Barking and Dagenham). We did not have data from 2018/19 from Barking and Dagenham, therefore we compared 2017/18 local data to 2018/19 CSDS data. We do not expect any significant changes in the distribution of IMD and ethnicity in such a short time frame (one year). To determine whether the high correlation group was representative of the whole of CSDS, we compared the ethnicity and IMD in the high correlation group to all of CSDS for children aged less than 5 years.

Results

Identifying "high correlation" local authorities

Thirty-three local authorities met the high correlation criteria for research into the 2-2½ year review. These are listed in Box 1. These groupings do not reflect the quality of local data recording for case management purposes or the quality of the health visiting service. The groupings are based solely on the correlation between the reference data and the CSDS extract.

For peer review only

Box 1: Local authorities that had data in CSDS with high correlation between data in CSDS and reference data based on indicators in table 2 for 2018/19

Blackpool	Hertfordshire	Sefton
Calderdale	Islington	Shropshire
Cornwall and Isles of Scilly	Kingston upon Thames	Somerset
Coventry	Kirklees	Southampton
Cumbria	Knowsley	St Helens
Derby	Lincolnshire	Telford and Wrekin
Devon	Liverpool	Thurrock
Dudley	North Somerset	Walsall
Gloucestershire	Peterborough	Warrington
Hampshire	Portsmouth	Worcestershire
Haringey	Rochdale	York

Are children in the high correlation local authorities nationally representative?

Recording of children in CSDS varied by ethnicity but not deprivation. ONS births reported a higher percentage of births of White children compared to CSDS: 72% vs 61% (Table A2). However, the data from ONS births included Wales, which likely has a higher proportion of White children than England. The percentage of children who were Asian, Black or another ethnicity were similar, but 14% of children were missing ethnicity compared to 4% in ONS births. This suggests that White children in CSDS were more likely to have missing data for ethnicity. In East Sussex, White children were under-represented and children with mixed ethnicity were over-represented in CSDS, compared to local data. In Barking and Dagenham, the percentage of White children was similar in CSDS and local data, but the local data recorded more children with mixed ethnicity and fewer Black and Asian children. As the overall number of children was similar, this is likely due to differences in recording, rather than differences in which children are in the datasets. The distribution of IMD in CSDS was similar to ONS births and local data from East Sussex and Barking and Dagenham (Table A6).

Children in the high correlation group were not representative of all children in CSDS in terms of ethnicity. The percentage of children in the mixed, Asian, Black and other ethnicity categories was lower in the high correlation group compared to all of CSDS (Table A2). This likely reflects regional differences in ethnicity. People in the Black, Asian, mixed and other ethnic groups are more likely to live in London than any other region in England, but only three of 32 London boroughs are in the high correlation subset(31). Children in the high correlation group were similar in terms of deprivation.

For peer review only

Table A2: The distribution of ethnicity and IMD for children aged less than 5 in the high correlation group compared to all CSDS and children in ONS births compared to births in CSDS

	Children aged <5 years in 2018/19		Births	
	Children in the high correlation group (33 LAs) [1]	All children in CSDS (149 LAs)	Children in ONS births (2018) [2]	Children in CSDS born in 2018/19 (151 LAs)
**Ethnicity**				
White	297,365 (68%)	1,845,171 (56%)	468,456 (72%)	316,820 (61%)
Mixed	32,255 (7%)	325,145 (10%)	[3]	[3]
Asian	23,350 (5%)	298,071 (9%)	56,821 (8%)	48,265 (9%)
Black	9,865 (2%)	129,352 (4%)	27,363 (4%)	19,610 (4%)
Other	10,200 (2%)	109,987 (3%)	77,060 (12%)	59,880 (12%)
Missing	64,360 (15%)	609,939 (18%)	27,023 (4%)	75,375 (14%)
Total	437,395	3,314,665	656,723	519,950
**IMD**				
1 (most deprived)	112,305 (26%)	832,885 (25%)	161,709 (26%)	129,915 (25%)
2	88,285 (20%)	714,728 (22%)	140,416 (22%)	113,005 (22%)
3	85,145 (19%)	640,986 (19%)	121,500 (19%)	103,655 (20%)
4	76,630 (18%)	575,712 (17%)	107,339 (17%)	91,340 (18%)
5 (least deprived)	75,025 (17%)	550,354 (17%)	94,687 (15%)	82,030 (16%)
Total	437,390	3,314,665	625,651	519,945

[1] CSDS data for the high correlation LAs is rounded to the nearest 5 [2] Ethnicity includes children born in Wales which is why the total is higher; [3] ONS do not report mixed as an ethnicity category in ONS births, therefore we have grouped mixed with other for this comparison

For peer review only

Supplementary Material 2: Estimating the percentage of eligible children with a 2-2½ year review in CSDS

We were unable to determine time at risk or the exact number of children in CSDS who were eligible for the 2-2½ year review, due to the lack of a precise measure of age in our CSDS extract. The age variable available was age on 31st March 2019. Children eligible for the 2-2½ year review during 2018/19 could be aged 2 or 3 at the end of 2018/19, and we could not identify those children who received their review during 2017/18 or 2019/20.

To estimate the percentage of children who had a 2-2½ year review, we used the number of children who had the 2-2½ year review and were aged 2 (at end of 2018/19) as the numerator (Equation 1). To calculate the denominator, we used all children aged 2 (at the end of 2018/19) minus the number of children who had the 2-2½ year review aged 3 (at end of 2018/19). This was based on the assumptions that (i) children who had the 2-2½ year review and were aged 3 (at end of 2018/19) were born towards the end of the year (i.e. were younger than 2½ years at the start of 2018/19), and (ii) a similar number of children who were aged 2 (at end of 2018/19) would have not yet turned 2½ years and would be expected to have their 2-2½ year review in 2019/20. If we had not estimated the percentage in this way, we would have either only counted children who had their 2-2½ year review and were aged 2 at the end of 2018/19 (excluding children who were born late in the year and would have their review the following year), or included in the denominator all children aged 2 or 3 who had a record of 2-2½ year review (including 3 year olds who had their review in the previous year (2017/18) and 2 year olds who were not due their review until the following year (2019/20)).

Equation 1: Estimation of the percentage of children with a 2-2½ year review

$$\text{Percentage of children with a 2 - 2.5 year review} = \frac{\text{Children aged 2 with a 2 - 2.5 year review}}{(\text{Children aged 2}) - (\text{Children aged 3 with a 2 - 2.5 year review})}$$

Supplementary Material 3: Comparison of our results with experimental statistics from Public Health England: explanation of discrepancies

There are some slight discrepancies between our results and the experimental statistics published by PHE,(1) which report relative coverage of the 2-2½ year review in 2018/9 including by socioeconomic and demographic variables.

PHE has labelled its statistics as 'experimental', to be interpreted with caution and subject to further updates. This reflects the fact that PHE analyses were conducted rapidly, to support urgent policy needs with resources of 1 month of senior analyst time (personal communication with PHE, May 2021). Our analyses had a methodological focus, aiming to explore the completeness of CSDS data and how it might be best used for research. We used the 2-2½ year review as the substantive exemplar question for our foundational work on the quality and completeness of the CSDS data. Our study had 6 months of researcher time, supervisory support from two senior academic staff and input from the authorship team.

The summary below has been developed following conversations with colleagues at PHE

Discrepancies:

PHE report that children with a safeguarding vulnerability were slightly more likely to receive a 2-2½ year review than their peers were as we found the inverse.

PHE report that Looked after children were less likely to receive a 2-2½ year review than their peers which is consistent with our findings, but we found a greater difference than reported by PHE.

PHE report that children from ethnic minority groups were less likely to have their 2-2½ year review than children recorded as 'white'. In contrast we did not find any meaningful differences between the proportion of children in different ethnic groups who received their 2-2½ year review.

Probable explanation

1. local authorities included in analyses

Our research uses a research-ready subset of CSDS data from 33 local authorities with higher data completeness. For safeguarding vulnerabilities and Looked After Children analyses were restricted to 7 and 13 local authorities, respectively which had <10% missing data for these variables. PHE used data from all local authorities in England. AS part of our discussions with PHE, the analysts re-ran the anlayses on the 33 local authorities which we used, for comparison. Difference in sample is likely to explain the discrepancies in results for safeguarding vulnerabilities. When restricted to the 33 local authorities, PHE analyses also found that children with safeguarding vulnerabilities were slightly less likely to receive a review compared to their peers (OR 0.9). The difference in magnitude of results for Looked After Children (who were less likely to have a review in both sets of results) is likely due to differences in the local authorities included in the analyses. Our analyses might be less subject to misclassification of Looked After Children as analyses were restricted to local authorities with <10% missing data for this variable. There is also the possibility that the 13 local authorities with <10% missing data were systematically different from the 33 research ready local authorities and all local authorities in England.

The ethnic distribution of the research-ready dataset is different from the whole of CSDS, with the % of children in the mixed, Asian, Black and other categories lower in the research-ready dataset than in CSDS as a whole. This likely reflects regional differences in ethnicity. People in the Black, Asian, mixed and other ethnic groups are more likely to live in London than any other region in England, but only three London boroughs are in the high correlation subset. The PHE experimental statistics report substantial regional variation in the relative odds of receiving a 2-2½ year review for children in different ethnic groups. This means that any 'over-representation' of a region in the research-ready subset is likely to generate results substantially different from analyses of all local authorities in England. Alternatively, it might be that poor data quality in the entire CSDS compared to the research-ready subset has led to the difference in results.

2. Years of data and age of child

PHE used two years' worth of data and imputed data for children missing in one year who should appear in both. This imputation was not completed in our research, which only used data from 2018/9. PHE included data for aged 3, unlike our study, so may have picked up some 2-2.5 year reviews completed at age 3.

Note: the discrepancies in results for ethnicity are hardest to explain and persist even after PHE re-ran analyses on the 33 research ready local authorities used in the our analyses.

Conclusion:

Analyses of the CSDS data are sensitive to small changes in analytical approaches and sensitivity analyses should be carried out as routine.

References

1: Public Heath England, *Characteristics of children receiving Universal Health Visitor Reviews: Experimental analysis of the Community Services Dataset. Available at* https://www.gov.uk/government/publications/characteristics-of-children-receiving-universal-health-visitor-reviews *[last Accessed 25.02.21]*. 2021.

review only

STROBE 2007 (v4) Statement—Checklist of items that should be included in reports of *cross-sectional studies*

**Section/Topic**	**Item #**	**Recommendation**	**Reported on page #**
**Title and abstract**	1	(*a*) Indicate the study's design with a commonly used term in the title or the abstract	Title "cross-sectional analysis"
		(*b*) Provide in the abstract an informative and balanced summary of what was done and what was found	Abstract p. 2
**Introduction**			
Background/rationale	2	Explain the scientific background and rationale for the investigation being reported	Intro p.4-6
Objectives	3	State specific objectives, including any prespecified hypotheses	Aim p. 6
**Methods**			
Study design	4	Present key elements of study design early in the paper	Mention cross-sectional design and the administrative data source (CSDS) in the title. We also mention CSDS in the aims section p.6
Setting	5	Describe the setting, locations, and relevant dates, including periods of recruitment, exposure, follow-up, and data collection	**England** (abstract and methods) **CSDS administrative data** (mentioned in title, abstract and described fully in methods) **2018-19** (abstract, methods) **33 local authorities** (abstract and methods) **Number of children**

For peer review only

			**in the sample and sub-group analyses** (methods)
Participants	6	(*a*) Give the eligibility criteria, and the sources and methods of selection of participants	Brief description on p.7 and full details in supplementary material 1
Variables	7	Clearly define all outcomes, exposures, predictors, potential confounders, and effect modifiers. Give diagnostic criteria, if applicable	p. 8 in methods section
Data sources/ measurement	8*	For each variable of interest, give sources of data and details of methods of assessment (measurement). Describe comparability of assessment methods if there is more than one group	p. 8 in methods
Bias	9	Describe any efforts to address potential sources of bias	Using the research-ready subset of data, described p.7 and Supplementary material 1
Study size	10	Explain how the study size was arrived at	Children from local areas was sufficiently complete data in the national administrative dataset p.7
Quantitative variables	11	Explain how quantitative variables were handled in the analyses. If applicable, describe which groupings were chosen and why	p. 7 methods
Statistical methods	12	(*a*) Describe all statistical methods, including those used to control for confounding	We calculated % and average contacts, stratified by group (no modelling), p.7 methods
		(*b*) Describe any methods used to examine subgroups and interactions	We stratified by

For peer review only

			group (no modelling), p.7 methods
		(*c*) Explain how missing data were addressed	We assumed that missing data meant 'no event' for most variables (methods section) but limited our analyses to areas with the most complete data (p.7 methods). We describe the amount of missing data where we can and the implications for results/interpretation e.g. home visits p. 10
		(*d*) If applicable, describe analytical methods taking account of sampling strategy	n/a
		(*e*) Describe any sensitivity analyses	n/a
**Results**			
Participants	13*	(a) Report numbers of individuals at each stage of study—eg numbers potentially eligible, examined for eligibility, confirmed eligible, included in the study, completing follow-up, and analysed	Table 1 reports numerator and denominator for each analysis
		(b) Give reasons for non-participation at each stage	n/a
		(c) Consider use of a flow diagram	n/a
Descriptive data	14*	(a) Give characteristics of study participants (eg demographic, clinical, social) and information on exposures and potential confounders	Table 1
		(b) Indicate number of participants with missing data for each variable of interest	'Blank' data items were interpreted as 'no event' e.g. no

For peer review only

			record of a contact is interpreted as no contact (methods section)
Outcome data	15*	Report numbers of outcome events or summary measures	Table 1
Main results	16	(*a*) Give unadjusted estimates and, if applicable, confounder-adjusted estimates and their precision (eg, 95% confidence interval). Make clear which confounders were adjusted for and why they were included	Table 1 (no modelling, just unadjusted estimates)
		(*b*) Report category boundaries when continuous variables were categorized	n/a
		(*c*) If relevant, consider translating estimates of relative risk into absolute risk for a meaningful time period	n/a
Other analyses	17	Report other analyses done—eg analyses of subgroups and interactions, and sensitivity analyses	n/a
**Discussion**			
Key results	18	Summarise key results with reference to study objectives	Discussion p. 10
Limitations	19	Discuss limitations of the study, taking into account sources of potential bias or imprecision. Discuss both direction and magnitude of any potential bias	'Limitations' section p. 12
Interpretation	20	Give a cautious overall interpretation of results considering objectives, limitations, multiplicity of analyses, results from similar studies, and other relevant evidence	Implications and conclusions section,
Generalisability	21	Discuss the generalisability (external validity) of the study results	" Our results cannot be confidently generalised to all children in England" p.10
**Other information**			
Funding	22	Give the source of funding and the role of the funders for the present study and, if applicable, for the original study on which the present article is based	p.13

*Give information separately for cases and controls in case-control studies and, if applicable, for exposed and unexposed groups in cohort and cross-sectional studies.

For peer review only

Note: An Explanation and Elaboration article discusses each checklist item and gives methodological background and published examples of transparent reporting. The STROBE checklist is best used in conjunction with this article (freely available on the Web sites of PLoS Medicine at http://www.plosmedicine.org/, Annals of Internal Medicine at http://www.annals.org/, and Epidemiology at http://www.epidem.com/). Information on the STROBE Initiative is available at www.strobe-statement.org.

For peer review only

Title: Variation in health visiting contacts for children in England: cross-sectional analysis of the 2-2½ year review using administrative data (Community Services Dataset, CSDS)

Journal:	*BMJ Open*
Manuscript ID	bmjopen-2021-053884.R1
Article Type:	Original research
Date Submitted by the Author:	17-Dec-2021
Complete List of Authors:	Fraser, Caroline; UCL Great Ormond Street Institute of Child Health Population Policy and Practice<br>Harron, Katie; UCL Great Ormond Street Institute of Child Health Population Policy and Practice<br>Barlow, Jane; University of Oxford, Department of Social Policy and Intervention,<br>Bennett, Samantha; Kent Community Health NHS Foundation Trust<br>Woods, Geoffrey; East Sussex County Council, Early Help Data & Information Team<br>Shand, Jenny; UCL Partners, ; UCLPartners<br>Kendall, Sally; University of Kent,<br>Woodman, Jenny; UCL, Social Research Institute
**Primary Subject Heading**:	Public health
Secondary Subject Heading:	Paediatrics, Nursing, Health policy, Health services research, Epidemiology
Keywords:	PUBLIC HEALTH, Organisation of health services < HEALTH SERVICES ADMINISTRATION & MANAGEMENT, Child protection < PAEDIATRICS, Community child health < PAEDIATRICS

I, the Submitting Author has the right to grant and does grant on behalf of all authors of the Work (as defined in the below author licence), an exclusive licence and/or a non-exclusive licence for contributions from authors who are: i) UK Crown employees; ii) where BMJ has agreed a CC-BY licence shall apply, and/or iii) in accordance with the terms applicable for US Federal Government officers or employees acting as part of their official duties; on a worldwide, perpetual, irrevocable, royalty-free basis to BMJ Publishing Group Ltd ("BMJ") its licensees and where the relevant Journal is co-owned by BMJ to the co-owners of the Journal, to publish the Work in this journal and any other BMJ products and to exploit all rights, as set out in our licence.

The Submitting Author accepts and understands that any supply made under these terms is made by BMJ to the Submitting Author unless you are acting as an employee on behalf of your employer or a postgraduate student of an affiliated institution which is paying any applicable article publishing charge ("APC") for Open Access articles. Where the Submitting Author wishes to make the Work available on an Open Access basis (and intends to pay the relevant APC), the terms of reuse of such Open Access shall be governed by a Creative Commons licence – details of these licences and which Creative Commons licence will apply to this Work are set out in our licence referred to above.

Other than as permitted in any relevant BMJ Author's Self Archiving Policies, I confirm this Work has not been accepted for publication elsewhere, is not being considered for publication elsewhere and does not duplicate material already published. I confirm all authors consent to publication of this Work and authorise the granting of this licence.

For peer review only

Title: Variation in health visiting contacts for children in England: cross-sectional analysis of the 2-2½ year review using administrative data (Community Services Dataset, CSDS)

Authors

Dr Caroline Fraser, Research Fellow, UCL Great Ormond Street Institute of Child Health caroline.fraser@ucl.ac.uk

Dr Katie Harron, Associate Professor of Statistics, Population, Policy & Practice Dept, UCL Great Ormond Street Institute of Child Health. London. WC1N 1EH, UK k.harron@ucl.ac.uk

Professor Jane Barlow, Professor of Evidence Based Intervention and Policy Evaluation, Department of Social Policy and Intervention, University of Oxford OX1 2ER jane.barlow@spi.ox.ac.uk

Mrs Samantha Bennett, Consultant in Public Health, Kent Community Health NHS Foundation Trust, Trinity House, Eureka Park, Ashford TN25 4AZ samantha.bennett15@nhs.net

Mr Geoffrey Woods, Information Analyst, Early Help Data & Information Team, East Sussex County Council , Lewes, East Sussex, BN7 1UE geoffrey.woods@eastsussex.gov.uk

Dr Jenny Shand, Director, UCLPartners; Researcher, UCL; Care City Cohort Director, Care City W1T 7HA jenny.shand@uclpartners.com

Professor Sally Kendall, Professor of Community Nursing and Public Health, Centre for Health Services Studies, University of Kent, Canterbury, Kent, CT2 7NF s.kendall-608@kent.ac.uk

**Dr Jenny Woodman, Associate Professor of Child and Family Policy, UCL Social Research Institute, London, WC1H 00A j.woodman@ucl.ac.uk

**Corresponding author

Word count: 4101

Key words:

Health visitor; child; Health Services, Preventive; healthcare disparities

For peer review only

ABSTRACT

Objective

The 2-2½ year universal health visiting review in England is a key timepoint for assessing child development and promoting school readiness. We aimed to ascertain which children were least likely to receive their 2-2½ year review and whether there were additional non-mandated contacts for children who missed this review.

Design, setting, participants

Cross-sectional analysis of the 2-2½ year review and additional health visiting contacts for 181,130 children aged two in England 2018/9, stratified by ethnicity, deprivation, safeguarding vulnerability indicator and Looked After Child status.

Analysis

We used data from 33 local authorities submitting highly complete data on health visiting contacts to the Community Services Dataset (CSDS). We calculated the percentage of children with a recorded 2-2½ year review and/or any additional health visiting contacts and average number of contacts, by child characteristic.

Results

The most deprived children were slightly less likely to receive a 2-2½ year review than the least deprived children (72% v 78%) and Looked After Children much less likely, compared to other children (44% v 69%). When all additional contacts were included, the pattern was reversed (deprivation) or disappeared (Looked After children). A substantial proportion of all children (24%), children with a 'safeguarding vulnerability' (22%) and Looked After children (29%) did not have a record of either a 2-2½ year review or any other face-to-face contact in the year.

Conclusions

A substantial minority of children aged 2 with known vulnerabilities did not see the health visiting team at all in the year. Some higher need children (e.g. deprived and Looked After) appeared to be seeing the health visiting team but not receiving their mandated health review. Further work is needed to establish the reasons for this, and potential solutions. There is an urgent need to improve the quality of national health visiting data.

For peer review only - http://bmjopen.bmj.com/site/about/guidelines.xhtml

ARTICLE SUMMARY: strengths and limitations of this study

- This is the first study to analyse the coverage and intensity of health visiting in England, also taking into account additional (non-mandated) contacts from the health visiting team.
- We addressed incompleteness in the national administrative data on health visiting in England (CSDS) by limiting our analyses to subsets of most complete data by a) developing methods to identify a research-ready subset of the national data using comparisons to reference data sources b) limiting analyses to local areas with <10% missing data for vulnerability indicators.
- Our approach to dealing with incomplete data (including only most complete data) limits the generalisability of our results to the whole of England, with particular implications for results about vulnerable children and different ethnic groups
- We were reliant on the information recorded in the administrative data, all of which is entered by the health visiting teams
- Despite the limitations, this is an important contribution to the evidence-base about how health visiting is delivered in England, which is foundational to making any improvements or modifications to the service.

For peer review only

INTRODUCTION

In England, there has been a sustained cross-government focus on identifying services and policies for babies and young children to reduce inequalities.(1-5) These policies represent a response to the evidence that at age 5 years, certain groups of children are so far behind in terms of development that they will "struggle to ever catch up".(6) Children who start school with lower than expected levels of development are more likely to be excluded from school or have social services involvement by the time they are 11.(6) By age 16, disadvantaged children are 18 months behind their peers and 40% of this development gap had already emerged by the age of five.(6)

There is one universal intervention programme for preschool children in England, which specifically aims to address inequalities and promote health and development of young children: The Healthy Child Programme (HCP 0-5, see Fig 1), led by Health Visitors. Health visitors are specialist child and family public health nurses who lead a team of mixed skill staff (see Fig 1). There are models of health visiting in some countries of the world: Child Health Nurses (Sweden), Public Health Nurses (America Canada, Ireland), Child and Family Health Nurses (Australia), Plunket Nurses (New Zealand), Social Nurses (Belgium), Lady Health Visitors and Lady Health Workers (Pakistan) and Patronage Nurses (Serbia, Kosovo, Kazakhstan). A However, health Visiting in England is unique in its universal coverage (Fig 1). A key part of the HCP 0-5 is five mandated contacts by the health visiting team before a child turns 3 years old (Fig 1). The last of these reviews is at 2-2½ years, and represents a vital opportunity to assess a child's readiness to learn, their physical and social and emotional development and to identify any additional support needed to start school on a level footing with their peers.(6, 7) Since 2018, there has been particularly high cross-government policy focus on the role that the 2-2½ year review can play in reducing social inequalities in cognitive development.(8-10) As part of a holistic assessment of the child during the 2-2½ year review, practitioners are required to use the Ages and Stages Questionnaire (ASQ-3™ (11)), and since 2020, the Early Language Identification Measure (ELIM) is recommended. See Fig 1 for a more detailed explanation of these tools.

There is now extensive recognition that the critical 1001 days (conception to age 2) represents the best opportunity for intervening with the aim of reducing inequalities, and the Leadsom report (March 2021) highlights the need for every local authority to develop a Best Start for Life offer, in order to achieve this.(10) To evaluate the impact of specific policies and interventions delivered in the critical 1001 days, we need a measure of child development for all children at the end of this period (age 2) which is complete, accurate and available for analysis, such as the ASQ-3 and/or ELIM.

For peer review only - http://bmjopen.bmj.com/site/about/guidelines.xhtml

Despite recognition of the importance of the 2-2½ year review both for supporting individual families and for collecting data to support planning and policies at a local and national population level, there is evidence that a substantial proportion of children still do not receive a 2-2½ year review. Data from Public Health England (PHE) 'interim reporting metrics' (referred to as 'metrics' in this paper) indicate that 22% of eligible children in England did not have a record of 2-2½ year review in 2018-20, with substantial variation across the country (27-97%).(6, 12) PHE has now been replaced with Office for Health Improvement and Disparities (OHID, 2021) so we refer to (ex)PHE. The Children's Commissioner and (ex)PHE both estimate that 9 out of 10 children receiving a 2-2½ year review also had an ASQ-3 completed but other studies have found significant variability across England in the implementation and reporting of the ASQ-3.(13-15)

The sub-universal reach of the 2-2½ year review has prompted questions about whether some children are systematically more likely to miss out on a review. In 2020, the Children's Commissioner tried to ascertain if vulnerable children were differentially likely to have a 2-2½ year review than their peers but found that most local authorities did not collect the necessary data, concluding: "There is little evidence that local areas are ensuring that their vulnerable young children are checked".(13) Since then, (ex)PHE has published experimental statistics (16), suggesting that the likelihood of receiving mandated reviews varies with ethnicity, and that children living in the most affluent areas of England were more likely to receive a mandated review than children living in the more deprived areas.(10) However, these emerging social patterns need further confirmation. Additionally, we do not know whether additional (non-mandated) contacts by the health visiting team are similarly patterned. It may be, for example, that the children who miss out on a mandated review are seeing members of the health visiting team regularly for other reasons. More recent experimental statistics from (ex)PHE (June 2021) suggested that children in the most deprived neighbourhoods are more likely to get additional contacts from the health visiting, but these analyses did not also include the mandated reviews.(17, 18)

AIM

We aimed to ascertain whether certain groups of children were less likely to receive their 2-2½ year review than other children. We used a national administrative dataset (the Community Services Dataset; CSDS (17)) to calculate the percentage of children in 2018/9 who received their 2-2½ year review, stratified by ethnic group, deprivation quintile, safeguarding vulnerability and Looked After Child Status. We investigated whether those that missed out on their 2-2½ year review were seeing the health visiting team for other reasons in the same time period.

For peer review only - http://bmjopen.bmj.com/site/about/guidelines.xhtml

METHODS

Study design and setting

This study comprises a cross-sectional analysis of coverage of the 2-2½ year health visiting review and coverage and intensity of additional health visiting contacts for children aged two in England in 2018/9, stratified by ethnic group, deprivation and 'vulnerability' indicators.

Data Source

The CSDS contains individual-level longitudinal administrative data from community services in England since 2015, including data on mandated and additional contacts with health visiting services. It is operated by NHS Digital, and providers of publicly-funded community services (including NHS trusts, private providers and the voluntary sector) are legally mandated to submit data. CSDS captures basic child characteristics (age, ethnicity), contacts with health visiting services (type, frequency, length, date) and a wider range of identified needs in children such as referral from or to specialist services. Quintiles of deprivation are derived from the Index of Multiple Deprivation, based on the child's postcode of usual address.

CSDS is the only child-level national source of information about health visiting in England but is a relatively new dataset, with outputs classified by NHS Digital as experimental. To our knowledge, the only published analysis of CSDS has been (ex)PHE's experimental statistics describing patterns in mandated reviews, cited by the Leadsom review (10, 16) and (separately) patterns of additional contacts.(17)

Our 'research-ready' subset of data

We analysed a pseudonymised extract of CSDS that was held within (ex)PHE for the purpose of delivering (ex)PHE's core work programme and priorities, for contacts between 1 April 2018 and 31 March 2019. To account for variation in the completeness of CSDS data across England and over time, we developed and applied methods for identifying a subset of 'research-ready' CSDS data for analysis (full details in Supplementary Material 1). The research-ready subset of data was restricted to LAs with a high level of data completeness. We checked the completeness of CSDS by comparing the number of eligible children and health visitor contacts recorded in CSDS with ONS data on births, health visitor contacts reported within (ex)PHE metrics, and anonymised health visiting data obtained directly from three LAs.

For peer review only

Our research-ready dataset for analyses of the 2-2½ year review in 2018/9 included 181,130 children from 33 local authorities. Children in the research-ready dataset were similarly deprived but less ethnically diverse than all children in England (see Supplementary Material 1). We identified a further subset of 13 local authorities with 18,240 children with sufficiently complete data (<10% missing) for analyses of Looked After Child Status and 7 local authorities with 15,485 children with sufficiently complete data on the data item "Safeguarding Vulnerability".

Analyses

We estimated the percentage of eligible children who received a 2-2½ year review. Our denominator comprised all children aged 2 on 31 March 2019 minus the number of children who had the 2-2½-year review and were aged 3 on 31 March 2019 (see Supplementary Material 2 for full details). The numerator comprised children aged 2 on 31 March 2019 who had a 2-2½ year review scheduled and coded as 'attended' or with missing attendance data (5% of all 2-2½ year reviews).

We calculated the percentage of children who received any face-to-face contact with a member of the heath visiting team for any reason (including the mandated 2-2½ year review and additional contacts) in 2018/9, by location of contact (home/any other location). We calculated the median and interquartile range for the number of all attended contacts per child (including letters and phone calls) and face-to-face 'attended' contacts. We quantified the number of children with a recorded ASQ-3 but no 2-2½ year review recorded, as an indication of 2-2½ year reviews that took place but might not have a 2-2½ year review code attached.

We stratified results by ethnicity, deprivation (IMD quintile) and vulnerability, where a 'vulnerable' child was defined as one with a code indicating a safeguarding vulnerability or Looked After Child at any point during 2018/19. Both codes are entered manually by a member of the health visiting team as part of their usual care of the child. The 'safeguarding vulnerability' code indicates factors such as referral from police or children's social care, significant injury, known or suspected domestic abuse, worrying parent behaviour or concerns about parental mental health. (19) Analysis of these characteristics was only conducted for LAs with less than 10% missing data for these variables (N=13 for Looked After Children and N=7 for safeguarding vulnerability). As IMD was complete for all 33 LAs and ethnicity had a similar level of completeness (16-23% missing) across all LAs, we included 33 LAs in analyses of these variables.

Patient and Public Involvement

We did not conduct any patient or public involvement for this study.

For peer review only - http://bmjopen.bmj.com/site/about/guidelines.xhtml

RESULTS

In our research-ready dataset, 74% of eligible children received their 2-2½ year review, 76% had any face-to-face contact with the health visiting team in the previous 12 months (including a 2-2½ year review and additional contacts) and 78% had *any* contact, including letters or phone calls (Table 1). If we assume that **all** the children with an ASQ-3 record had a 2-2½ year review, our estimate of children with a 2-2½ year review increases to 81%.

The most deprived quintile of children were less likely to have received a 2-2½ year review (72%) than children in the least deprived quintile (78%), with a gradient across quintiles. This pattern was reversed (on a small scale) when additional contacts were taken into account (80% for the most deprived v 78% for the least deprived quintile, Table 1). Looked After Children were much less likely to have received a 2-2½ year review recorded compared to other children (44% compared to 69%, Table 1). However, this difference disappeared when all face-to-face additional contacts with the health visiting team were included. The lower proportion of 2-2½ year reviews for Looked After Children was not explained by missed appointments: all 90 Looked After Children with scheduled 2-2½ year reviews were recorded as 'attended'.

The small number of children with a safeguarding vulnerability (n=205), were less likely to have received a 2-2½ year review (83%) than other children (86%) and this held true when additional contacts were taken into account. Three were no clear patterns of 2-2½ year reviews across ethnic groups (Table 1). However, analyses that included additional contacts showed there was a lower proportion of white children receiving contacts (73%) than children in the other ethnic categories (80-90%, Table 1).

The majority of all children (76%), children with safeguarding vulnerabilities recorded (78%) and Looked After children (71%), had a record of a contact with health visiting services, either for a 2-2½ year review or as an additional contact (Table 1). The most common face-to-face activities (n=110,780) for children aged 2, other than the 2-2½ year review, were “other” (31%, n=34,180), assessments (23%, n=26,015), counselling, advice or support (17%, n=18,875) and clinical interventions (15%, n=17,145), which include individualised care plans, for example parenting advice, support with behaviour and child development. These figures include multiple activities recorded for the same child (including on the same day).

A substantial proportion of all children (24%), children with a ‘safeguarding vulnerability’ recorded (22%) and Looked After children (29%) did not have a record of either a 2-2½ year review or any other face-to-face contact (Table 2). Most of the children without a face-to-face contact did not have

For peer review only - http://bmjopen.bmj.com/site/about/guidelines.xhtml

a record of letters or telephone calls from health visiting services, suggesting no contacts were attempted. On average, children that had any type of contact with health visiting services saw a member of the team once in this period, compared to 3 times for children with a safeguarding vulnerability code and 5 times for children with a Looked After Child code (Table 2).

For peer review only

For peer review only - http://bmjopen.bmj.com/site/about/guidelines.xhtml

Table 1: Percentage of children aged 2 with a 2-2½ year review or any contact (face-to-face or other medium) with a health visiting team

Child characteristic	With a 2-2½ year review attended		Children aged 2 on 31 March 2019 with any contact with health visiting teams			
			Face-to-face contacts		Any (including letters and telephone)	
	% of eligible* children (95% CI)	Number with a review / eligible children	% of eligible* children (95% CI)	Number with a review / eligible children	% of eligible* children (95% CI)	Number with a review / eligible children
**All children (33 LAs)**	74% (73%, 74%)	55,975 / 75,960	76% (75%, 76%)	70,695 / 93,525	78% (78%, 78%)	73,065 / 93,525
**Safeguarding vulnerability code (7 LAs)**						
No	86% (85%, 86%)	13,090 / 15,280	80% (80%, 81%)	14,550 / 18,150	83% (83%, 84%)	15,120 / 18,150
Yes	83% (78%, 88%)	170 / 205	78% (73%, 83%)	195 / 250	80% (75%, 85%)	200 / 250
**Looked After child (13 LAs)**						
No	69% (69%, 70%)	19,475 / 28,035	77% (77%, 78%)	26,820 / 34,705	79% (78%, 79%)	27,355 / 34,705
Yes	44% (37%, 51%)	90 / 205	71% (65%, 77%)	170 / 240	73% (67%, 79%)	175 / 240
**Quintile of IMD (33 LAs)**						
1 (most deprived)	72% (72%, 73%)	14,295 / 19,800	78% (78%, 79%)	18,620 / 23,785	81% (80%, 82%)	19,305 / 23,785
2	72% (71%, 72%)	11,065 / 15,475	76% (76%, 77%)	14,420 / 18,910	79% (78%, 79%)	14,870 / 18,910
3	73% (72%, 74%)	10,685 / 14,680	74% (73%, 74%)	13,630 / 18,465	76% (75%, 77%)	14,035 / 18,465
4	75% (75%, 76%)	9,850 / 13,075	73% (73%, 74%)	11,985 / 16,345	76% (75%, 77%)	12,420 / 16,345
5 (least deprived)	78% (77%, 78%)	10,045 / 12,940	76% (75%, 76%)	12,165 / 16,015	78% (78%, 79%)	12,565 / 16,015
**Ethnicity (33 LAs)**						
White	73% (72%, 73%)	33,455 / 45,920	73% (72%, 73%)	42,360 / 58,150	75% (75%, 75%)	43,655 / 58,150
Mixed	79% (78%, 80%)	5,875 / 7,405	90% (89%, 91%)	7,390 / 8,210	91% (91%, 92%)	7,505 / 8,210
Asian or Asian British	76% (75%, 78%)	2,920 / 3,825	80% (78%, 81%)	3,555 / 4,470	81% (80%, 82%)	3,620 / 4,470
Black or Black British	73% (71%, 75%)	1,185 / 1,620	80% (79%, 82%)	1,530 / 1,905	82% (80%, 83%)	1,555 / 1,905
Other	63% (61%, 66%)	1,045 / 1,650	71% (69%, 73%)	1,450 / 2,030	75% (73%, 77%)	1,520 / 2,030

LAs: Local Authorities. IMD: Index of Multiple Deprivation. *Note: The percentage of children aged 2 with any face-to-face contact may be lower than the percentage of children with a 2-2½ year review because we reduced the number of children in the denominator for the 2-2½ year review to account for children with the 2-2½ year review aged 3 (based on age at end of 2018/19), and a small number of 2-2½ year reviews were not be face-to-face (see Supplementary Material 2 for full explanation).

For peer review only

Table 2: Median contacts per child for children aged 2 with any recorded contact during 2018/19

**Child characteristic**	**Median (interquartile range) number of contacts aged 2 for children with contacts**	
	**Any contact**	**Face-to-face**
**All children (33 LAs)**	1 (1 to 2)	1 (1 to 2)
**Safeguarding factors (7 LAs)**		
No	1 (1 to 2)	1 (1 to 2)
Yes	3 (2 to 7)	3 (1 to 7)
**Looked After child (13 LAs)**		
No	1 (1 to 2)	1 (1 to 2)
Yes	6 (3 to 10)	5 (3 to 9)
**Quintile of IMD (33 LAs)**		
1 (most deprived)	2 (1 to 4)	2 (1 to 3)
2	2 (1 to 3)	1 (1 to 3)
3	1 (1 to 3)	1 (1 to 2)
4	1 (1 to 2)	1 (1 to 2)
5 (least deprived)	1 (1 to 2)	1 (1 to 2)
**Ethnicity (33 LAs)**		
White	1 (1 to 3)	1 (1 to 2)
Mixed	1 (1 to 3)	1 (1 to 2)
Asian or Asian British	2 (1 to 2)	1 (1 to 3)
Black or Black British	2 (1 to 4)	1 (1 to 3)
Other	1 (1 to 3)	1 (1 to 2)

LAs: Local Authorities. IMD: Index of Multiple Deprivation.

For peer review only

Overall, 28% of children with a face-to-face contact were seen at home, and the percentage of at home contacts was greater for Looked After children (63%), children with safeguarding vulnerabilities (78%), and children living in the most deprived areas (38%) (Table 3). However, if all contacts with missing data on location (12%; 8805/70,695) were in fact home visits, the proportion of all face-to-face contacts that were in a child's home could be as high as 40%.

For peer review only

Table 3: Percentage of children aged 2 with a record of a face-to-face contact in any location and at home by child characteristic

Child characteristic	Children aged 2 with a record of a face-to-face contact [1]			
	In any location		At home	
	% (95% CI)	Children with a contact / children aged 2	% (95% CI)	Children with a contact / children aged 2
**All children (33 LAs)**	76% (75%, 76%)	70,695 / 93,525	28% (27%, 28%)	26,130 / 93,525
**Quintile of IMD (33 LAs)**				
1 (most deprived)	78% (78%, 79%)	18,620 / 23,785	38% (37%, 38%)	8,920 / 23,785
2	76% (76%, 77%)	14,420 / 18,910	28% (27%, 29%)	5,345 / 18,910
3	74% (73%, 74%)	13,630 / 18,465	27% (26%, 27%)	4,900 / 18,465
4	73% (73%, 74%)	11,985 / 16,345	24% (23%, 24%)	3,845 / 16,345
5 (least deprived)	76% (75%, 76%)	12,165 / 16,015	20% (19%, 21%)	3,210 / 16,015
**Ethnicity (33 LAs)**				
White	73% (72%, 73%)	42,360 / 58,150	27% (27%, 28%)	15,985 / 58,150
Mixed	90% (89%, 91%)	7,390 / 8,210	25% (24%, 26%)	2,020 / 8,210
Asian or Asian British	80% (78%, 81%)	3,555 / 4,470	32% (31%, 33%)	1,435 / 4,470
Black or Black British	80% (79%, 82%)	1,530 / 1,905	32% (31%, 33%)	595 / 1,905
Other	71% (69%, 73%)	1,450 / 2,030	31% (29%, 33%)	630 / 2,030
**Looked After children (13 LAs)**				
No	77% (77%, 78%)	26,820 / 34,705	27% (27%, 28%)	9,500 / 34,705
Yes	71% (65%, 77%)	170 / 240	63% (56%, 67%)	150 / 240
**Safeguarding factors (7 LAs)**				
No	80% (80%, 81%)	14,550 / 18,150	38% (38%, 39%)	6,420 / 18,150
Yes	78% (73%, 83%)	195 / 250	65% (59%, 70%)	130 / 205

[1] Rounded to nearest 5 to reduce risk of disclosure as data is subnational; LAs: Local Authorities; IMD: Index of Multiple Deprivation

For peer review only - http://bmjopen.bmj.com/site/about/guidelines.xhtml

DISCUSSION

Main findings

The majority (>70%) of eligible children in our sample, including those defined as vulnerable, were engaged with health visiting services and received their 2-2½ year review. Children in the most deprived areas were *slightly* more likely to miss out on a 2-2½ year review and Looked After children were *much* more likely to miss out on this review. However, when all additional contacts were included, the pattern was reversed (for deprivation) or disappeared (for Looked After children). This highlights the importance of including additional contacts in analyses when investigating whether the delivery of health visiting in England is socially patterned. We also found that Looked After and the most deprived children received more intensive health visiting than their peers, with more frequent face-to-face contacts. Although we found no evidence that children with a safeguarding vulnerability recorded were any more or less likely to receive a 2-2½ year review or an additional contact than their peers, we did find that when this group had at least one face-to-face contact, they received more intensive health visiting than average.

Our results suggest that there are groups of high need children (e.g. deprived children and Looked After children) who are engaged with the health visiting team but do not receive their mandated 2-2½ year review. We could not ascertain how far children without a 2-2½ year review were having their needs formally assessed through other routes such as a statutory Looked After Child review, as part of a Child Protection Plan or had developmental delay or additional needs identified by the health visiting team prior to the 2-2 ½ year review, as suggested by an analysis by the Children's Commissioner in 2020.(13) The same analysis by the Children's Commissioner reported that some local authorities had stated that the 2-2 ½ year review may not always be appropriate when reviewing children with complex heath needs or development delays.(13) Our findings raise similar questions about whether the 2-2½ year review and/or the ASQ-3 is perceived as appropriate for high need groups in the population, both by professionals and parents/carers.

Our results also suggest that there might be two distinct groups of vulnerable children with regard to the receipt of health visiting. We identified a majority group of vulnerable children who received multiple face-to-face contacts within a 12-month period, often in the child's home. This group appears to be receiving intensive health visiting, consistent with the model of proportionate universalism that underpins health visiting in England (a universal service for all children but with greater support and service provision for children in families with identified needs). The second group comprised a substantial minority of vulnerable children who were not in contact with the health visitor team at all: 22% of children with safeguarding vulnerabilities and 29% of Looked After

For peer review only - http://bmjopen.bmj.com/site/about/guidelines.xhtml

children did not have a record of either a 2-2½ year review or any other face-to-face additional contact in the year. As we did not find evidence of attempted contacts with these children in CSDS, there is a real possibility that these vulnerable children might not have heard from or seen a member of the health visiting team within the year.

Our results on the 2-2½ year review are largely consistent with the experimental statistics published by (ex)PHE, with the exception of ethnicity.(16) (Ex)PHE found that children from ethnic minority groups were less likely than white children to receive the mandated reviews, including the 2-2½ year review.(16) The difference is likely due to poor data completeness in CSDS and different analytical approaches to dealing with this incompleteness (see supplementary material 3). Our results on additional contacts are also largely consistent with the (ex)PHE experimental statistics, which suggest that children likely to have higher needs (e.g. deprived children and receiving statutory child protection services) receive more additional contacts than average.(17) Our results build on the (ex)PHE statistics by bringing together mandated and additional contacts in one analysis, which facilitates for the first time estimates of children not seeing the health visiting team for any reason in the 12 month period.

Implications

Our data indicates that the health visiting service fell short of universal reach for children aged 2 years in England in 2018/9. Together, our results and the (ex)PHE experimental statistics suggest that 2-2½ year reviews are socially patterned. However, the scale of the difference and whether it exists for different ethnic groups remains uncertain, largely as a result of poor data completeness in CSDS (supplementary material 3). Poor data remains a significant barrier to understanding how health visiting is currently delivered, and the available national data for England can only generate results which should be treated as hypotheses, with a need for further exploration.

Although the health visiting service seems to be achieving a model of proportionate universalism for **some** children with known vulnerabilities (with multiple visits a year, often in the child's home), there was also a substantial minority of vulnerable children without any recorded contact from the health visiting team. This is of significant concern, given the likely concentration of health and welfare need in these groups and makes it unlikely that the current format and delivery of the 2-2½ year review is helping to address inequalities in school readiness as intended. The secondary effects of the COVID-19 pandemic, including heightened need and reduced workforce capacity (particularly the number of qualified health visitors), are likely to exacerbate the shortfall both in universal reach and provision of extra intensive services where needed.(20)

For peer review only - http://bmjopen.bmj.com/site/about/guidelines.xhtml

Conclusions

Since completion of this study, the HCP is being revised to be 'universal in reach and personalised in response'.(21) Our study evidences the gap between this policy ambition and the current reality of health visiting.(22) Further work is needed to establish reasons for low coverage of the 2-2½ year review in Children Looked After and to generate recommendations about the most suitable way of delivering the 2-2½ year review to a) all children and b) Looked After Children and other groups with known complex needs or vulnerabilities. Relevant factors may include skill mix (health visitor versus other team member, see Fig 1), continuity of care, quality of reviews and referral pathways following identification of need.

In order to produce reliable estimates, researchers require access to high quality national data from health visiting services linked to other datasets (such as primary care, hospital, social care and education) for the whole of England.(23) CSDS is not yet sufficiently complete.(24) NHS Digital and (ex)PHE have been undertaking quality improvement work to address this. The findings of this study suggest that policies and/or funding to accelerate the pace of CSDS quality improvements should be considered at a national level. Further work is needed both on how best to support local practitioners to improve data recording in their systems and on how data transfer from local systems into CSDS might be improved, given the evidence that some data on health visiting contacts in local systems do not make it into CSDS.(24)

Limitations

Our data did not allow us to follow a child over time, which might mean we slightly underestimate 2-2½ year reviews as some children may have had their 2-2½ year review just before or after our study period. As administrative data, CSDS codes will not capture everything health visitors identify or deliver. Information might be coded with high accuracy on local systems but 'lost' in the transfer to NHS Digital, e.g. with use of slightly different codes. There is some evidence of this, based on our comparison of two local areas with CSDS data.(24) Second, contacts may be miscoded. Although our analyses of ASQ-3 records provide some evidence of this, the consistency of our results with other data (24) suggests that miscoding does not explain the 1 in 5 children 'missing' a 2-2½ year review. We know that much abuse and neglect of children will not be coded in administrative data, even if it is suspected by professionals (25, 26) which will lead to under-estimating differences between vulnerable and other children. Many data items were too poorly completed to use, for example child disability and staff type (e.g. health visitor, nursery nurse). CSDS does not capture quality or meaning of the interaction between health visitors and parents. To obtain a full picture of health visiting

For peer review only - http://bmjopen.bmj.com/site/about/guidelines.xhtml

activity in England, and the contribution of this service to child health and wellbeing, all types of LAs need to be investigated using a triangulation of data sources, including CSDS, locally held data, surveys and in-depth qualitative data collected from professionals and families, as a study in Scotland is attempting(27) and as we have funding to do for children living with adverse childhood experiences in England.(28)

Our results cannot be confidently generalised to all children in England. The children in the 'research-ready' data were not nationally representative in terms of ethnicity (Supplementary Material 1) and the excluded LAs might be systematically different from those we included which had more complete data. Although our findings should be treated as hypotheses, this study marks an important first step in making use of existing, routinely collected data to understand the coverage and intensity of health visiting services in England.

FUNDING

This study/project was supported by the National Institute for Health Research (NIHR) Policy Research Programme (PR-PRU-1217-21301). The views expressed are those of the author(s) and not necessarily those of the NIHR, the Department of Health and Social Care or Public Health England.

This project is delivered as part of (ex)PHE's 2020/21 Life Course Intelligence business plan for child and maternal health in order to understand CSDS data quality and its suitability for further analysis to understand trends, variation and inequalities in health visiting and outcomes for children in the early years. An honorary contract with (ex)PHE enabled the researcher to work as part of the analytical team and analyse the CSDS for this purpose.

This research was supported in part by the NIHR Great Ormond Street Hospital Biomedical Research Centre and the Health Data Research UK (grant No. LOND1), which is funded by the UK Medical Research Council and eight other funders. KH is supported by funding from NIHR (17/99/19).

AUTHOR CONTRIBUTIONS

JW and KH conceived of the study, were responsible for design of the study and supervised all analyses which were carried out by CF. JW, KH, CF, JB, SB, GW and JS all contributed substantially to the design of the study. CF, KH, JB, SB, GW, JS, SK and JW substantially contributed to interpretation of data. JS, GW, SB provided access to locally held data and gave expert advice on data and the service context. CF and JW drafted the paper and KH, JB, SB, GW, JS and SK substantially contributed to its intellectual content, including in multiple revisions. CF, KH, JB, SB, GW, JS, SK and JW approve

For peer review only

the final version of the paper for publication. JW and KH are jointly responsible for the overall content as guarantors

ACKNOWLEDGEMENTS

We would like to thank individuals in Public Health England for their comments and advice on early drafts, including Wendy Nicholson within Nursing, Maternity and Early years and Kate Thurland and Helen Smith. We thank Professor Ruth Gilbert at UCL Great Ormond Street Institute of Child Health for input into study design, methods and drafts. We are grateful to Care City, Barking and Dagenham, Havering and Redbridge CCG, London Borough of Barking and Dagenham, East Sussex County Council and East Sussex Healthcare Trust and Kent County Council and Community Health NHS Foundation Trust for access to their local data and knowledge and expertise. We thank individuals at the Institute of Health Visiting for comments on this paper and earlier drafts.

COMPETING INTERESTS

None of the authors have any competing interests to declare.

DATA AVAILABILITY

The authors do not have permission for onward sharing of the individual-level data underlying this article. Requests to access the CSDS data can be made to NHS Digital. The study was approved by UCL Institute of Education Research Ethics Committee on 01.05.20 (Ref 1333).

Figure Legends

Figure 1: Delivery of the Health Child Programme (HCP) by Health Visiting Teams and use of ASQ-3 and ELIM in the 2-2½ year review

For peer review only

References

1. Gov.uk/news. New focus on babies' and children's health as review launches. Available at https://www.gov.uk/government/news/new-focus-on-babies-and-childrens-health-as-review-launches [last accessed 28.09.20] 2020.

2. Marmot M, Goldblatt P, Allen J. Fairer society, Healthy Lives (the Marmot review). London: The Marmot Review 2010.

3. A cross-party manifesto. The 1001 Critical Days: the importance of the conception to age 2 period. London: Wave Trust and NSPCC 2014. Available at https://www.wavetrust.org/1001-critical-days-the-importance-of-the-conception-to-age-two-period [last accessed 29.09.20]..

4. Early Years and Family Support Ministerial Group. First 1000 Days of Life. Available at https://publications.parliament.uk/pa/cm201719/cmselect/cmhealth/1496/149602.htm [last accessed 12.12.21] 2018.

5. National Health Service. The NHS Long Term Plan. London: NHS 2019.

6. Office for the Children's Commissioner. Best Beginnings in the Early Years: A proposal for a new early years guarantee to give all children in England the best start in life. London: OCC 2020.

7. Department for Health. Universal Health Visitor Reviews: advice for local authorities in delivery of the mandated universal health visitor reviews from 1 October 2015. London: DH 2015.

8. Public Health England. Early language identification measure and intervention. Available at https://assets.publishing.service.gov.uk/government/uploads/system/uploads/attachment_data/file/939872/ELIM_Handbook_December-2020.pdf [Last accessed 09.02.21]. 2020.

9. Public Health England, Department of Health and Social Care. Early language identification measure and intervention: guidance handbook. Available at https://assets.publishing.service.gov.uk/government/uploads/system/uploads/attachment_data/file/939872/ELIM_Handbook_December-2020.pdf [last accessed 10.05.21]. 2020.

10. HM Government. The Best Start for Life: a vision for the 1,001 Critical Days. The Early Years Healthy Development Review Report. London: Her Majesty's Stationery Office 2021.

11. Squires J, Brickers D, Twombley E, Nickel R, Clifford J, Murphy K. Ages & Stages Questionnaires®, Third Edition (ASQ-3™): A Parent -Completed Child Monitoring System. Baltimore: Paul H. Brookes Publishing Co., Inc.; 2009.

12. Public Health England. Health visitor service delivery metrics: 2018 to 2019. Available at https://www.gov.uk/government/statistics/health-visitor-service-delivery-metrics-2018-to-2019 [last accessed 12.12.21] 2019.

13. Office for the Children's Commissioner. Best beginnings in the early years: Technical report two. London: OCC 2020.

14. Kendall S, Nasg A, Braun A, Bastug G, Rougeux E, Bedford H. Evaluating the use of a population measure of child development in the Healthy Child Programme Two Year Review.

For peer review only

Available at https://kar.kent.ac.uk/54584/1/evaluating-the-use-of-a-population-measure-of-child-development-in-the-healthy-child-two-year-review.pdf [last accessed 09.02.21]. 2014.

15. Kendall S, Nash A, Braun A, Bastug G, Rougeaux E, Bedford H. Acceptability and understanding of the Ages & Stages Questionnaires(R), Third Edition, as part of the Healthy Child Programme 2-year health and development review in England: Parent and professional perspectives. Child Care Health Dev. 2019;45(2):251-6.

16. Public Heath England. Characteristics of children receiving Universal Health Visitor Reviews: Experimental analysis of the Community Services Dataset. Available at https://www.gov.uk/government/publications/characteristics-of-children-receiving-universal-health-visitor-reviews [last Accessed 25.02.21]. 2021.

17. P Public Health England. Contacts other than those mandated and referrals by health visitors - Experimental analysis of the Community Services Dataset. Available at Contacts other than those mandated and referrals by health visitors - GOV.UK (www.gov.uk) [Last Accessed 17.06.21] 2021

18. Woodman J, Harron K, Hancock D. Which children in England see the health visiting team and how often? Journal of Health Visiting. 2021;9(7):282-4.

19. NHS Digital. Community Services Data Set technical output specification 2018 [Available from: https://digital.nhs.uk/data-and-information/data-collections-and-data-sets/data-sets/community-services-data-set/community-services-data-set-technical-output-specification. [last accessed 15.12.21]

20. Barlow J, Bach-Mortensen A, Homonchuk O, Woodman J. The impact of the COVID-19 pandemic on services from pregnancy through age 5 years for families who are high risk of poor outcomes or who have complex social needs. Available at https://www.ucl.ac.uk/children-policy-research/sites/children-policy-research/files/the_impact_of_the_covid-19_pandemic_on_services_from_pregnancy_through_age_5_years_final_report_101120.pdf [Last Accessed 11.02.21]. 2020.

21. Department of Health and Social Care. Advancing our health: prevention in the 2020s (consultation). Available at www.gov.uk/government/consultations/advancing-our-health-prevention-in-the-2020s [accessed 11.12.19]. 2019.

22. Bryar RM, Cowley DS, Adams CM, Kendall S, Mathers N. Health visiting in primary care in England: a crisis waiting to happen? Br J Gen Pract. 2017;67(656):102-3.

23. ADRUK webpage. ECHILD: Linking children's health and education data for England. Available at https://www.adruk.org/our-work/browse-all-projects/echild-linking-childrens-health-and-education-data-for-england-142/ [Last Accessed 11.02.21] 2021 [

24. Fraser C, Harron K, Barlow J, Bennett S, Woods G, Shand J, et al. How can we use the community services dataset (CSDS) for research into health visiting? Available at https://www.ucl.ac.uk/children-policy-research/sites/children-policy-research/files/using_csds_for_research_report_08.10.20.pdf [last accessed 14.01.21]. 2020.

For peer review only

25. Woodman J, Allister J, Rafi I, de Lusignan S, Belsey J, Petersen I, et al. A simple approach to improve recording of concerns about childmaltreatment in primary care records: developing a quality improvement intervention. British Journal of General Practice. 2012;62(600):e478-e86.

26. Woodman J, Hodson D, Gardner R, Cuthbert C, Woolley A, Allister J, et al. The GP's role in responding to child maltreatment. London: NSPCC; 2014.

27. Doi L, Morrison K, Astbury R, Eunson J, Horne MA, Jepson R, et al. Study protocol: a mixed-methods realist evaluation of the Universal Health Visiting Pathway in Scotland. BMJ open. 2020;10(12):e042305.

28. NIHR. Funding and Awards. 2021. Available at https://fundingawards.nihr.ac.uk/award/NIHR129901 [last accessed 12.12.21]

REFERENCES FOR FIGURE 1

1. Public Health England. Healthy Child Programme: The 4-5-6 approach for health visiting and school nursing. 2018.
2. Department for Health. Healthy Child Programme. Pregnancy and the First Five Years. London; 2009.
3. Conti G, Dow A. Using FOI data to assess the state of Health Visiting Services in England before and during COVID-19. London; 2021.
4. Office for the Children's Commissioner. Best beginnings in the early years: Technical report two. Available at https://www.childrenscommissioner.gov.uk/wp-content/uploads/2020/07/cco-best-beginnings-in-the-early-years-tech-report-2.pdf [last accessed 10.02.21]. 2020.
5. Institute of Health Visiting. State of Health Visiting in England: we need more health visitors. 2021.
6. Institute of Health Visiting. Health visting in England: a vision for the future. London; 2019.
7. Department of Health. Universal Health Visitor Reviews: advice for local authorities in delivery of the mandated universal health visitor reviews from 1 October 2015. London; 2015.
8. Public Health England. Best start in life and beyond: Improving public health outcomes for children, young people and families. Guidance to support the commissioning of the Healthy Child Programme 0-19: Health Visiting and school nursing services. Commissioning guide 2. London; 2018.
9. Squires J, Bricker D, Twombley E. Ages & stages questionnaires: Social-emotional (Vol. 2). Baltimore: Paul H. Brookes Publishing Company.; 2002.
10. Sheldrick RC, Marakovitz S, Garfinkel D, Carter AS, Perrin EC. Comparative Accuracy of Developmental Screening Questionnaires. JAMA pediatrics. 2020;174(4):366-74.
11. NHS Digital, Ofsted. Ages and Stages Questionnaire (ASQ-3) Analysis Children and Young People's Health Services Data Set (CYPHS), Experimental Statistics, October 2016 - March 2017. Available at https://assets.publishing.service.gov.uk/government/uploads/system/uploads/attachment_data/file/643759/ASQ3_analysis_Oct16_to_Mar17_main_findings.pdf (last accessed 11.02.21]. 2017.
12. Kendall S, Nasg A, Braun A, Bastug G, Rougeux E, Bedford H. Evaluating the use of a population measure of child development in the Healthy Child Programme Two Year Review. Available at https://kar.kent.ac.uk/54584/1/evaluating-the-use-of-a-population-measure-of-child-development-in-the-healthy-child-two-year-review.pdf [last accessed 09.02.21]. 2014.
13. Kendall S, Nash A, Braun A, Bastug G, Rougeaux E, Bedford H. Acceptability and understanding of the Ages & Stages Questionnaires(R), Third Edition, as part of the Healthy Child

For peer review only

Programme 2-year health and development review in England: Parent and professional perspectives. Child Care Health Dev. 2019;45(2):251-6.
14. Public Health England. Early language identification measure and intervention. Available at https://assets.publishing.service.gov.uk/government/uploads/system/uploads/attachment_data/file/939872/ELIM_Handbook_December-2020.pdf [Last accessed 09.02.21]. 2020.
15. Public Health England. Identifying and supporting children's early language needs. Summary report. Available at https://assets.publishing.service.gov.uk/government/uploads/system/uploads/attachment_data/file/939833/ELIM_Summary_report_December-2020.pdf [Last accessed 26.05.21]. 2020.
16. Department for Education. Unlocking talent, fulfilling potential. A Plan for improving social mobility in England. Available at https://assets.publishing.service.gov.uk/government/uploads/system/uploads/attachment_data/file/667690/Social_Mobility_Action_Plan_-_for_printing.pdf [last accessed 26.05.21]. 2017.
17. Public Health England, Department of Health and Social Care. Early language identification measure and intervention: guidance handbook. Available at https://assets.publishing.service.gov.uk/government/uploads/system/uploads/attachment_data/file/939872/ELIM_Handbook_December-2020.pdf [last accessed 10.05.21]. 2020.

For peer review only

Delivery of the Health Child Programme (HCP) by Health Visitors

The Healthy Child Programme (HCP) is designed to promote the health and development of young children, and prevent and mitigate the impact of adversity and inequalities in early childhood.(1, 2). The HCP is led by health visitors: registered nurses or midwives who have undertaken an additional Specialist Community Public Health Nursing qualification. Health visiting teams comprise health visitors with a specialist qualification in public health and other clinical skill mix staff such as band 5 staff nurses and nursery nurses. There is wide variation in the skill mix within health visiting teams: in February 2020 health visitors made up on average 70% of teams, but there was substantial variation across local areas, with a range from 33% to 100%.(3) Studies report that a minority (between a third (4)and a fifth (31)) of 2-2½ year reviews are conducted by qualified health visitors in England and that 21% of heath visitors report that families in their local area always received this review from a qualified health visitor (5). As part of the HCP, LAs are required to commission delivery of a minimum of five contacts for every child and family in England before the child is aged 5 years in which health visitors or other members of the team review the child's health and development in the context of family health and environment, offer support in a range of areas, and signpost to community resources such as children's centres, child care and wider early years services.(6) The health visiting contacts can be delivered through home visits, individual or group clinic appointments, or phone calls and are supported by administrative activity such as letters to families.(7) The five mandated reviews, also known as 'universal health reviews', are an antenatal visit after 28 weeks of pregnancy, a new birth visit within 14 days, a 6 to 8 week review, an age one year review and a 2-2½ year review.(1)

Ages and Stage Questionnaire (ASQ-3)

The ASQ-3 is a tool to measure child development. Policy guidance for England states that the ASQ-3 should always be used as part of the 2-2½ year review (8) and practitioners might also choose to use the ASQ-SE™, social and emotional questionnaire.(9) In 2020 the Office for Children's Commissioner in England estimated that 9 in 10 children with a 2-2½ review had a ASQ-3 assessment. (4) ASQ as a screening tool for developmental delay in children <5y delivered by Paediatricians has been compared to other screening tools in American populations and judged to have "modest sensitivity" for detecting developmental delay but "adequate specificity" (i.e. they missed lots of children with developmental delay but didn't pick up too many false positives). (10) There is no equivalent study for ASQ-3 as delivered by health visitors and nursery nurses in an English setting, though an analysis done by Ofsted and NHS Digital (2017) reported that scores on ASQ-3 used at the 2-2½ year review were not well correlated at either a national or local level with the Early Years Foundation Stage Profile (an developmental assessment routinely completed for every child by their teacher at the end of the first year of school).(11) The lack of studies validating ASQ-3 as an individual level screening tool for developmental delay in an English setting drives the English policy guidance that ASQ-3 should be used as a *population measure* of child development for monitoring and to inform interventions, not as an individual assessment or screening tool. (8) However, a study in 4 sites in England (2014) found confusion among parents and health care professionals about the purpose of ASQ-3, namely whether it was a tool for assessing a child's individual development or a population measure of child development(12). In 2020 the Office for the Children's Commissioner for England found evidence that ASQ-3 was being used in a large number of local authorities as a screening tool.(4) The 2014 study in 4 sites in England concluded that variation in the use of ASQ (e.g. sometimes completed by professionals and sometimes by parents) potentially undermines its usefulness as a population measure of child development (13)

Early Language Identification Measure (ELIM)

ELIM-E is a tool designed for use in the 2-2½ year review in order to identify children with speech, communication or learning needs and which should be used as part of a local pathway to identify and support individual children towards school readiness and, ultimately, reduce inequalities in school readiness across the population.(14, 15) The Early Language Identification Measure (ELIM) and accompanying intervention was commissioned in 2018 as part of the UK government's Social Mobility Action Plan,(16) with guidance issued to commissioners, service leads and practitioners in late 2020.(15, 17) Unlike the ASQ-3 tool, Public Health England and Department of Health and Social Care recommend the use of ELIM-E for assessing need in individual children.

For peer review only

Supplementary Material 1: Identifying a 'research-ready' subset of CSDS for use in research into the 2-2½ year review

The material in supplementary material 1 and 2 is based on our report: https://www.ucl.ac.uk/children-policy-research/sites/children-policy-research/files/using_csds_for_research_report_08.10.20.pdf

Methods

Identifying "high correlation" data for local authorities

To identify local authorities within CSDS with sufficiently complete data for research into 2-2½ year reviews, we created two indicators of how well data was recorded in CSDS and sense-checked these indicators with a Consultant in Public Health working with a Health Visiting Service (SB), an information analyst who manages health visiting data (GW) and the lead for PHE metrics (KT). The indicators measure the agreement between CSDS and aggregate reference data (PHE metrics or ONS births). Based on these indicators, we categorised local authorities as having high, moderate or low correlation between data in CSDS and reference data.

The indicators are based on the number of eligible children and children who had 2-2½ year reviews recorded (Table A1). These indicators were selected to account for the two mechanisms of under-recording in CSDS that would affect research into health visiting activity. First, some children may be missing completely from the data. If children are missing from CSDS, we do not have accurate data on how many children are eligible for health visiting and therefore do not have an accurate denominator to calculate the percentage of children who received contacts. We were able to determine whether the number of eligible children were accurate through comparisons between CSDS and ONS birth statistics. We focused on "eligible children" who were aged 0 because we did not have reliable reference data on the number of children aged 2 or 3 to compare with CSDS. Second, contacts with health visitors may be under-recorded. We were able to determine whether the number of contacts were accurate through comparisons between CSDS and PHE metrics.

Table A1: The indicators and limits for identifying high or moderate correlation local authorities in CSDS

Indicator	Limits	
	High correlation	Moderate correlation
**(i) Eligible children:** Agreement between the number of children aged 0 at the end of the reporting year in CSDS and ONS birth statistics	- 10% to +20%	-30% to +30%
**(ii) 2-2.5 year reviews:** Agreement between the number of children with a 2-2.5 year review in CSDS and PHE metrics	-15% to +15%	-40% to +40%

We used local data from East Sussex and Kent to select appropriate limits for the high and moderate correlation categories, by comparing local data to the reference data. The percentage difference between the local data and PHE metrics ranged from -10% to +1% for the new birth visit and 2-2½ year review (See Table A1). Based on this, we considered a difference between CSDS and PHE

For peer review only

metrics of +/-15% to be appropriate for high correlation and +/-40% appropriate for moderate correlation. We expect the true number of "eligible children" (children born in 2018/19) to be higher than the number of children born in England, because CSDS should include children born elsewhere who moved to England before their first birthday. This was true in our comparisons of local data and ONS births, where there were 3-8% more children aged <1 year in the local data compared to ONS births. This is reflected in the limits we selected for high correlation for eligible children (-10% to +20%).

Local authorities that did not meet either high or moderate criteria were classified as low correlation. As for many local authorities agreement with the indicators varied by quarter, we allowed local authorities to move between categories by quarter. This means that data from specific time periods within a local authority could be considered as high correlation and therefore included in analyses. The contact date was used to allocate children who had a contact to a local authority quarter (and therefore to a correlation category). For children without contacts (i.e. to calculate the denominator population), we could not determine which child became eligible in each quarter. Therefore, we calculated the total number of children per local authority divided by four to estimate the number of children per quarter.

Are children in the high correlation local authorities nationally representative?

To draw conclusions from research using the subset of children in the high correlation group we must understand whether results can be extrapolated to children in other local authorities in CSDS, and to all children whether or not they were recorded in CSDS. To determine whether the subset of local authorities were representative, we focused on the IMD quintile and ethnicity because of the completeness of these variables in CSDS (IMD is complete for all children in the study and ethnicity is complete for 82% of children) and because there were a substantial number of children within each IMD or ethnicity category.

To determine whether the chances of a child being recorded in CSDS varied by IMD quintile and ethnicity, we compared the distribution of IMD and ethnicity in ONS births to children in CSDS who were born in 2018/19. We also compared the distribution of IMD quintile and ethnicity of children aged <5 years in CSDS and local data for two local authorities (East Sussex and Barking and Dagenham). We did not have data from 2018/19 from Barking and Dagenham, therefore we compared 2017/18 local data to 2018/19 CSDS data. We do not expect any significant changes in the distribution of IMD and ethnicity in such a short time frame (one year). To determine whether the high correlation group was representative of the whole of CSDS, we compared the ethnicity and IMD in the high correlation group to all of CSDS for children aged less than 5 years.

Results

Identifying "high correlation" local authorities

Thirty-three local authorities met the high correlation criteria for research into the 2-2½ year review. These are listed in Box 1. These groupings do not reflect the quality of local data recording for case management purposes or the quality of the health visiting service. The groupings are based solely on the correlation between the reference data and the CSDS extract.

For peer review only

Box 1: Local authorities that had data in CSDS with high correlation between data in CSDS and reference data based on indicators in table 2 for 2018/19

Blackpool	Hertfordshire	Sefton
Calderdale	Islington	Shropshire
Cornwall and Isles of Scilly	Kingston upon Thames	Somerset
Coventry	Kirklees	Southampton
Cumbria	Knowsley	St Helens
Derby	Lincolnshire	Telford and Wrekin
Devon	Liverpool	Thurrock
Dudley	North Somerset	Walsall
Gloucestershire	Peterborough	Warrington
Hampshire	Portsmouth	Worcestershire
Haringey	Rochdale	York

Are children in the high correlation local authorities nationally representative?

Recording of children in CSDS varied by ethnicity but not deprivation. ONS births reported a higher percentage of births of White children compared to CSDS: 72% vs 61% (Table A2). However, the data from ONS births included Wales, which likely has a higher proportion of White children than England. The percentage of children who were Asian, Black or another ethnicity were similar, but 14% of children were missing ethnicity compared to 4% in ONS births. This suggests that White children in CSDS were more likely to have missing data for ethnicity. In East Sussex, White children were under-represented and children with mixed ethnicity were over-represented in CSDS, compared to local data. In Barking and Dagenham, the percentage of White children was similar in CSDS and local data, but the local data recorded more children with mixed ethnicity and fewer Black and Asian children. As the overall number of children was similar, this is likely due to differences in recording, rather than differences in which children are in the datasets. The distribution of IMD in CSDS was similar to ONS births and local data from East Sussex and Barking and Dagenham (Table A6).

Children in the high correlation group were not representative of all children in CSDS in terms of ethnicity. The percentage of children in the mixed, Asian, Black and other ethnicity categories was lower in the high correlation group compared to all of CSDS (Table A2). This likely reflects regional differences in ethnicity. People in the Black, Asian, mixed and other ethnic groups are more likely to live in London than any other region in England, but only three of 32 London boroughs are in the high correlation subset(31). Children in the high correlation group were similar in terms of deprivation.

For peer review only

Table A2: The distribution of ethnicity and IMD for children aged less than 5 in the high correlation group compared to all CSDS and children in ONS births compared to births in CSDS

	Children aged <5 years in 2018/19		Births	
	Children in the high correlation group (33 LAs) [1]	All children in CSDS (149 LAs)	Children in ONS births (2018) [2]	Children in CSDS born in 2018/19 (151 LAs)
**Ethnicity**				
White	297,365 (68%)	1,845,171 (56%)	468,456 (72%)	316,820 (61%)
Mixed	32,255 (7%)	325,145 (10%)	[3]	[3]
Asian	23,350 (5%)	298,071 (9%)	56,821 (8%)	48,265 (9%)
Black	9,865 (2%)	129,352 (4%)	27,363 (4%)	19,610 (4%)
Other	10,200 (2%)	109,987 (3%)	77,060 (12%)	59,880 (12%)
Missing	64,360 (15%)	609,939 (18%)	27,023 (4%)	75,375 (14%)
Total	437,395	3,314,665	656,723	519,950
**IMD**				
1 (most deprived)	112,305 (26%)	832,885 (25%)	161,709 (26%)	129,915 (25%)
2	88,285 (20%)	714,728 (22%)	140,416 (22%)	113,005 (22%)
3	85,145 (19%)	640,986 (19%)	121,500 (19%)	103,655 (20%)
4	76,630 (18%)	575,712 (17%)	107,339 (17%)	91,340 (18%)
5 (least deprived)	75,025 (17%)	550,354 (17%)	94,687 (15%)	82,030 (16%)
Total	437,390	3,314,665	625,651	519,945

[1] CSDS data for the high correlation LAs is rounded to the nearest 5 [2] Ethnicity includes children born in Wales which is why the total is higher; [3] ONS do not report mixed as an ethnicity category in ONS births, therefore we have grouped mixed with other for this comparison

For peer review only

Supplementary Material 2: Estimating the percentage of eligible children with a 2-2½ year review in CSDS

We were unable to determine time at risk or the exact number of children in CSDS who were eligible for the 2-2½ year review, due to the lack of a precise measure of age in our CSDS extract. The age variable available was age on 31st March 2019. Children eligible for the 2-2½ year review during 2018/19 could be aged 2 or 3 at the end of 2018/19, and we could not identify those children who received their review during 2017/18 or 2019/20.

To estimate the percentage of children who had a 2-2½ year review, we used the number of children who had the 2-2½ year review and were aged 2 (at end of 2018/19) as the numerator (Equation 1). To calculate the denominator, we used all children aged 2 (at the end of 2018/19) minus the number of children who had the 2-2½ year review aged 3 (at end of 2018/19). This was based on the assumptions that (i) children who had the 2-2½ year review and were aged 3 (at end of 2018/19) were born towards the end of the year (i.e. were younger than 2½ years at the start of 2018/19), and (ii) a similar number of children who were aged 2 (at end of 2018/19) would have not yet turned 2½ years and would be expected to have their 2-2½ year review in 2019/20. If we had not estimated the percentage in this way, we would have either only counted children who had their 2-2½ year review and were aged 2 at the end of 2018/19 (excluding children who were born late in the year and would have their review the following year), or included in the denominator all children aged 2 or 3 who had a record of 2-2½ year review (including 3 year olds who had their review in the previous year (2017/18) and 2 year olds who were not due their review until the following year (2019/20)).

Equation 1: Estimation of the percentage of children with a 2-2½ year review

$$\text{Percentage of children with a 2-2.5 year review} = \frac{\text{Children aged 2 with a 2-2.5 year review}}{\text{(Children aged 2) - (Children aged 3 with a 2-2.5 year review)}}$$

Supplementary Material 3: Comparison of our results with experimental statistics from Public Health England: explanation of discrepancies

There are some slight discrepancies between our results and the experimental statistics published by PHE,(1) which report relative coverage of the 2-2½ year review in 2018/9 including by socioeconomic and demographic variables.

PHE has labelled its statistics as 'experimental', to be interpreted with caution and subject to further updates. This reflects the fact that PHE analyses were conducted rapidly, to support urgent policy needs with resources of 1 month of senior analyst time (personal communication with PHE, May 2021). Our analyses had a methodological focus, aiming to explore the completeness of CSDS data and how it might be best used for research. We used the 2-2½ year review as the substantive exemplar question for our foundational work on the quality and completeness of the CSDS data. Our study had 6 months of researcher time, supervisory support from two senior academic staff and input from the authorship team.

The summary below has been developed following conversations with colleagues at PHE

Discrepancies:

PHE report that children with a safeguarding vulnerability were slightly more likely to receive a 2-2½ year review than their peers were as we found the inverse.

PHE report that Looked after children were less likely to receive a 2-2½ year review than their peers which is consistent with our findings, but we found a greater difference than reported by PHE.

PHE report that children from ethnic minority groups were less likely to have their 2-2½ year review than children recorded as 'white'. In contrast we did not find any meaningful differences between the proportion of children in different ethnic groups who received their 2-2½ year review.

Probable explanation

1. local authorities included in analyses

Our research uses a research-ready subset of CSDS data from 33 local authorities with higher data completeness. For safeguarding vulnerabilities and Looked After Children analyses were restricted to 7 and 13 local authorities, respectively which had <10% missing data for these variables. PHE used data from all local authorities in England. AS part of our discussions with PHE, the analysts re-ran the anlayses on the 33 local authorities which we used, for comparison. Difference in sample is likely to explain the discrepancies in results for safeguarding vulnerabilities. When restricted to the 33 local authorities, PHE analyses also found that children with safeguarding vulnerabilities were slightly less likely to receive a review compared to their peers (OR 0.9). The difference in magnitude of results for Looked After Children (who were less likely to have a review in both sets of results) is likely due to differences in the local authorities included in the analyses. Our analyses might be less subject to misclassification of Looked After Children as analyses were restricted to local authorities with <10% missing data for this variable. There is also the possibility that the 13 local authorities with <10% missing data were systematically different from the 33 research ready local authorities and all local authorities in England.

The ethnic distribution of the research-ready dataset is different from the whole of CSDS, with the % of children in the mixed, Asian, Black and other categories lower in the research-ready dataset than in CSDS as a whole. This likely reflects regional differences in ethnicity. People in the Black, Asian, mixed and other ethnic groups are more likely to live in London than any other region in England, but only three London boroughs are in the high correlation subset. The PHE experimental statistics report substantial regional variation in the relative odds of receiving a 2-2½ year review for children in different ethnic groups. This means that any 'over-representation' of a region in the research-ready subset is likely to generate results substantially different from analyses of all local authorities in England. Alternatively, it might be that poor data quality in the entire CSDS compared to the research-ready subset has led to the difference in results.

2. Years of data and age of child

PHE used two years' worth of data and imputed data for children missing in one year who should appear in both. This imputation was not completed in our research, which only used data from 2018/9. PHE included data for aged 3, unlike our study, so may have picked up some 2-2.5 year reviews completed at age 3.

Note: the discrepancies in results for ethnicity are hardest to explain and persist even after PHE re-ran analyses on the 33 research ready local authorities used in the our analyses.

Conclusion:

Analyses of the CSDS data are sensitive to small changes in analytical approaches and sensitivity analyses should be carried out as routine.

References

1: Public Heath England, *Characteristics of children receiving Universal Health Visitor Reviews: Experimental analysis of the Community Services Dataset. Available at* https://www.gov.uk/government/publications/characteristics-of-children-receiving-universal-health-visitor-reviews *[last Accessed 25.02.21]*. 2021.

STROBE 2007 (v4) Statement—Checklist of items that should be included in reports of *cross-sectional studies*

Section/Topic	Item #	Recommendation	Reported on page #
**Title and abstract**	1	(*a*) Indicate the study's design with a commonly used term in the title or the abstract	Title "cross-sectional analysis"
		(*b*) Provide in the abstract an informative and balanced summary of what was done and what was found	Abstract p. 2
**Introduction**			
Background/rationale	2	Explain the scientific background and rationale for the investigation being reported	Intro p.4-6
Objectives	3	State specific objectives, including any prespecified hypotheses	Aim p. 6
**Methods**			
Study design	4	Present key elements of study design early in the paper	Mention cross-sectional design and the administrative data source (CSDS) in the title. We also mention CSDS in the aims section p.6
Setting	5	Describe the setting, locations, and relevant dates, including periods of recruitment, exposure, follow-up, and data collection	**England** (abstract and methods) **CSDS administrative data** (mentioned in title, abstract and described fully in methods) **2018-19** (abstract, methods) **33 local authorities** (abstract and methods) **Number of children**

For peer review only

			**in the sample and sub-group analyses** (methods)
Participants	6	(*a*) Give the eligibility criteria, and the sources and methods of selection of participants	Brief description on p.7 and full details in supplementary material 1
Variables	7	Clearly define all outcomes, exposures, predictors, potential confounders, and effect modifiers. Give diagnostic criteria, if applicable	p. 8 in methods section
Data sources/ measurement	8*	For each variable of interest, give sources of data and details of methods of assessment (measurement). Describe comparability of assessment methods if there is more than one group	p. 8 in methods
Bias	9	Describe any efforts to address potential sources of bias	Using the research-ready subset of data, described p.7 and Supplementary material 1
Study size	10	Explain how the study size was arrived at	Children from local areas was sufficiently complete data in the national administrative dataset p.7
Quantitative variables	11	Explain how quantitative variables were handled in the analyses. If applicable, describe which groupings were chosen and why	p. 7 methods
Statistical methods	12	(*a*) Describe all statistical methods, including those used to control for confounding	We calculated % and average contacts, stratified by group (no modelling), p.7 methods
		(*b*) Describe any methods used to examine subgroups and interactions	We stratified by

For peer review only

			group (no modelling), p.7 methods
		(*c*) Explain how missing data were addressed	We assumed that missing data meant 'no event' for most variables (methods section) but limited our analyses to areas with the most complete data (p.7 methods). We describe the amount of missing data where we can and the implications for results/interpretation e.g. home visits p. 10
		(*d*) If applicable, describe analytical methods taking account of sampling strategy	n/a
		(*e*) Describe any sensitivity analyses	n/a
**Results**			
Participants	13*	(a) Report numbers of individuals at each stage of study—eg numbers potentially eligible, examined for eligibility, confirmed eligible, included in the study, completing follow-up, and analysed	Table 1 reports numerator and denominator for each analysis
		(b) Give reasons for non-participation at each stage	n/a
		(c) Consider use of a flow diagram	n/a
Descriptive data	14*	(a) Give characteristics of study participants (eg demographic, clinical, social) and information on exposures and potential confounders	Table 1
		(b) Indicate number of participants with missing data for each variable of interest	'Blank' data items were interpreted as 'no event' e.g. no

For peer review only

			record of a contact is interpreted as no contact (methods section)
Outcome data	15*	Report numbers of outcome events or summary measures	Table 1
Main results	16	(*a*) Give unadjusted estimates and, if applicable, confounder-adjusted estimates and their precision (eg, 95% confidence interval). Make clear which confounders were adjusted for and why they were included	Table 1 (no modelling, just unadjusted estimates)
		(*b*) Report category boundaries when continuous variables were categorized	n/a
		(*c*) If relevant, consider translating estimates of relative risk into absolute risk for a meaningful time period	n/a
Other analyses	17	Report other analyses done—eg analyses of subgroups and interactions, and sensitivity analyses	n/a
**Discussion**			
Key results	18	Summarise key results with reference to study objectives	Discussion p. 10
Limitations	19	Discuss limitations of the study, taking into account sources of potential bias or imprecision. Discuss both direction and magnitude of any potential bias	'Limitations' section p. 12
Interpretation	20	Give a cautious overall interpretation of results considering objectives, limitations, multiplicity of analyses, results from similar studies, and other relevant evidence	Implications and conclusions section,
Generalisability	21	Discuss the generalisability (external validity) of the study results	" Our results cannot be confidently generalised to all children in England" p.10
**Other information**			
Funding	22	Give the source of funding and the role of the funders for the present study and, if applicable, for the original study on which the present article is based	p.13

*Give information separately for cases and controls in case-control studies and, if applicable, for exposed and unexposed groups in cohort and cross-sectional studies.

For peer review only

Note: An Explanation and Elaboration article discusses each checklist item and gives methodological background and published examples of transparent reporting. The STROBE checklist is best used in conjunction with this article (freely available on the Web sites of PLoS Medicine at http://www.plosmedicine.org/, Annals of Internal Medicine at http://www.annals.org/, and Epidemiology at http://www.epidem.com/). Information on the STROBE Initiative is available at www.strobe-statement.org.

For peer review only
